# Elucidating the Role of Virulence Traits in the Survival of Pathogenic *E. coli* PI-7 Following Disinfection

**DOI:** 10.3389/fbioe.2020.614186

**Published:** 2020-12-22

**Authors:** Krishnakumar Sivakumar, Robert Lehmann, Andri Taruna Rachmadi, Nicolas Augsburger, Noor Zaouri, Jesper Tegner, Pei-Ying Hong

**Affiliations:** ^1^Computational Bioscience Research Center, Biological and Environmental Science and Engineering Division, King Abdullah University of Science and Technology, Thuwal, Saudi Arabia; ^2^Living Systems Laboratory, Environmental Epigenetic Program, Biological and Environmental Science and Engineering Division, Computer, Electrical and Mathematical Sciences and Engineering Division, King Abdullah University of Science and Technology, Thuwal, Saudi Arabia; ^3^Water Desalination and Reuse Center, Biological and Environmental Science and Engineering Division, King Abdullah University of Science and Technology, Thuwal, Saudi Arabia

**Keywords:** transposon mutagenesis, inactivation kinetics, chlorination, solar irradiation, UV 254

## Abstract

Reuse and discharge of treated wastewater can result in dissemination of microorganisms into the environment. Deployment of disinfection strategies is typically proposed as a last stage remediation effort to further inactivate viable microorganisms. In this study, we hypothesize that virulence traits, including biofilm formation, motility, siderophore, and curli production along with the capability to internalize into mammalian cells play a role in survival against disinfectants. Pathogenic *E. coli* PI-7 strain was used as a model bacterium that was exposed to diverse disinfection strategies such as chlorination, UV and solar irradiation. To this end, we used a random transposon mutagenesis library screening approach to generate 14 mutants that exhibited varying levels of virulence traits. In these 14 isolated mutants, we observed that an increase in virulence traits such as biofilm formation, motility, curli production, and internalization capability, increased the inactivation half-lives of mutants compared to wild-type *E. coli* PI-7. In addition, oxidative stress response and EPS production contributed to lengthening the lag phase duration (defined as the time required for exposure to disinfectant prior to decay). However, traits related to siderophore production did not help with survival against the tested disinfection strategies. Taken together, the findings suggested that selected virulence traits facilitate survival of pathogenic *E. coli* PI-7, which in turn could account for the selective enrichment of pathogens over the non-pathogenic ones after wastewater treatment. Further, the study also reflected on the effectiveness of UV as a more viable disinfection strategy for inactivation of pathogens.

## Introduction

Bacterial pathogens can be discharged into raw wastewater through fecal material, and despite going through the wastewater treatment processes, some tend to persist in treated wastewater effluent (Crockett, 2007; Shannon et al., 2007; Naidoo and Olaniran, 2014). The problem is further compounded if antibiotic resistant pathogenic strains survive the treatment process (Blasco et al., 2008; Munir et al., 2011) and are disseminated into the environment to potentially cause infection of living hosts (Naidoo and Olaniran, 2014; Adefisoye and Okoh, 2016). This highlights the requirement for effective strategies to mitigate the dissemination of fecal-borne pathogenic strains from wastewater to the natural environment.

Disinfection of secondary effluent from wastewater treatment plants (WWTPs) is typically proposed as a last treatment step based on the assumption that a strong oxidizing agent such as chlorine would effectively inactivate bacteria prior to discharge. Chlorine disinfects by oxidizing nucleic acids and damaging cell membranes as well as by generating hypochlorous acid and hypochlorite, which can induce oxidative stress to disrupt normal bacterial functions. However, chlorination also generates disinfection byproducts that can potentially stimulate natural transformation of extracellular DNA into competent bacterial cells (Mantilla-Calderon et al., 2019). UV has increasingly been considered as an alternative disinfection strategy, particularly for post-membrane bioreactor effluent since nucleic acids have high absorbance for UV at the 254 nm wavelength. Upon absorbance, UV causes dimerization of pyrimidines such as cytosine and thymine, which subsequently culminates in DNA damage and cell apoptosis (Dodd, 2012). Alternatively, low-cost treatment technologies like wetland or oxidation ponds make use of sunlight to further achieve biocidal effect through UV-A radiation and reactive oxygen species (ROS) (Ubomba-Jaswa et al., 2009; Augsburger et al., 2019). Few hours of exposure to simulated sunlight has resulted in rapid inactivation of fecal indicator organisms and waterborne pathogens (Wegelin et al., 1994; McGuigan et al., 1998; Boyle et al., 2008; Ubomba-Jaswa et al., 2009).

Despite the various disinfection strategies that have been put in place to mitigate bacterial contaminants in treated wastewater, pathogenic bacterial strains seem to be able to survive disinfection and remain viable in the final disinfected effluents. To emphasize, strains that have upregulated their virulence factors such as biofilm formation, siderophore production, curli, motility and internalization capability were reported to have survived tertiary treatment stages of WWTPs (Anastasi et al., 2013; Jumat et al., 2018). Our previous study also showed that the virulent and multidrug-resistant wastewater strain *E. coli* PI-7 (Mantilla-Calderon et al., 2016) required a longer sunlight exposure than commensal strain *E. coli* DSM 1103 prior to decaying, suggesting the increased ability of this pathogenic strain to survive solar irradiation (Al-Jassim et al., 2017).

Observations from these earlier studies suggest that virulence traits can potentially enhance the survival of bacterial strains to disinfection strategies such as chlorination, UV and sunlight irradiation. UV-irradiated pathogenic *E. coli* harboring virulence genes have shown tendency to undergo resuscitation from viable but not culturable state (VBNC) by sustaining its pathogenicity and physiological characteristics (Zhang et al., 2015). Ability of certain strains to undergo photoreactivation and self-healing DNA repair after UV treatment hinders the effectiveness of UV disinfection (Guo and Kong, 2019). Despite these observations, no systematic study has been conducted to assess which virulence traits harbored by these pathogens contribute to the elevated resistance toward disinfection strategies.

To elucidate the role of virulence traits in abating disinfection-facilitated decay, we used *E. coli* PI-7 as model system to construct a random transposon insertion site library using the Tn5 transposon. *E. coli* PI-7 harbors an extensive repertoire of virulence factors and other detoxification features such as heavy metal efflux pumps, UV protection enzymes, oxidative stress response and cellular repair factors (Mantilla-Calderon et al., 2016). Through screening of the random transposon insertion site library, we obtained a total of 14 mutants that displayed varying levels of H_2_O_2_-mediated oxidative stress response and virulence traits such as biofilm formation, siderophore generation, motility, internalization into mammalian cells relative to wild-type (WT) control. The mutants and WT strains were individually subjected to chlorination, UV and sunlight irradiation, and assessed for differences in their decay kinetics parameters such as lag phase and half-lives (determined post-lag phase). Mutants with differences in decay kinetic parameters relative to the control WT would imply that the virulence trait perturbed by transposon mutagenesis contributed a role toward resistance or susceptibility against disinfection. Findings from this study would clarify which specific virulence factors contribute to disinfection tolerance.

## Materials and Methods

### Construction of PI-7 Random Transposon Insertion Site Library

*E. coli* PI-7 is a pathogenic strain that was first isolated from a wastewater treatment plant in Jeddah, Saudi Arabia (Mantilla-Calderon et al., 2016). Transposon mutagenesis of *E. coli* PI-7 was conducted through mating PI-7 with *E. coli* harboring Tn5 (Auerswald et al., 1981; Jacobs et al., 2003), using protocols described from previous studies but with slight modifications (Thormann et al., 2004; Rizzi et al., 2008; Kouzuma et al., 2010; Ding et al., 2014). Briefly, overnight-propagated LB cultures of PI-7 and Tn5-harboring host strains were harvested, washed twice with 1× PBS (pH = 7.4) and then re-suspended in LB at a cell density equivalent to OD_600_∼1.0. Both recipient and host cells were then mixed together in volumetric ratio of 1:1, following which, 1 mL of the mixed culture was spotted on the center of an LB agar plate. The spotted mixed culture was incubated at 37°C for 6 h to facilitate conjugation. Post conjugation, the mixed culture were re-dissolved in LB and then spread on LB agar plates supplemented with 8 μg/mL meropenem, 80 μg/mL tetracycline and 200 μg/mL streptomycin, and then incubated at 37°C for 48 h to allow growth and observation of Tn5-inserted PI-7 transposon mutants. Tn5 transposon used in this study harbor genes encoding resistance to tetracycline and streptomycin and hence facilitate the selection of Tn5-inserted PI-7 transposon mutants, post conjugation. On the other hand, meropenem was added to ensure selection of colonies belonging to PI-7. Taken together, we constructed PI-7 random transposon insertion site library by applying selective antibiotic pressure of tetracycline and streptomycin on PI-7 at concentrations higher than minimum inhibitory concentration observed for PI-7 in our previous study (Mantilla-Calderon et al., 2016). Expanding the mutant selection window within spectrum higher than the MIC of tetracycline and streptomycin constituted the first round of screening Tn5-inserted PI-7 transposon mutants. A total of 200 transposon mutants were derived in this manner.

### Characterization of the PI-7 WT and Mutants

Genomic characterization of PI-7 has identified an extensive repertoire of virulence-based traits as well as an detoxification features such as multiple antibiotic resistance genes (ARGs) and heavy metal efflux pumps, UV protection enzymes, oxidative stress response and cellular repair factors (Mantilla-Calderon et al., 2016). Furthermore, PI-7 displayed upregulation of virulence factors while exhibiting relatively higher persistence against biocidal effects of solar radiation compared to *E. coli* DSM1103 (Al-Jassim et al., 2017). Hence, in order to elucidate the hypothesized role of virulence traits in enhancing resistance of PI-7 to disinfection strategies, the 200 mutants derived from the PI-7 random transposon insertion site library were further characterized primarily on the basis of virulence factors such as biofilm formation, EPS production, siderophore production, invasion on mammalian cells, curli production and motility. Mutants were also characterized on the basis of oxidative stress response to ascertain the contribution of virulence traits toward increasing oxidative stress response mechanisms. Since biofilm formation is the most important virulence factor toward pathogenic infections (O’Loughlin et al., 2013; Stêpień-Pyśniak et al., 2019) and survival strategy upon exposure to external stressors (Solano et al., 1998; Bonafonte et al., 2000; Al Safadi et al., 2012; Chua et al., 2015), mutants exhibiting higher (>1.5-times WT, *p* < 0.05) or lower (<0.75-times WT, *p* < 0.05) biofilm formation capability relative to WT were shortlisted for virulence characterization after the first round of screening. These mutants were compared against WT in terms of each virulence-based phenotypic trait. Details of phenotypic-based characterization of mutants are furnished in Supplementary Information 1.

Accordingly, 14 from the total of 200 isolated mutants was chosen for the study on the basis of their varied display in a broad spectrum of virulence factors relative to WT. Whole genome sequencing of these shortlisted mutants using PacBio Sequel platform was done by first extracting genomic DNA from PI-7 WT and mutants with a DNeasy blood and tissue kit (Qiagen, Hilden, Germany). Sequencing was then done on the DNA to identify the transposon mutagenesis-facilitated disruption sites in genes regulating virulence traits (Details in Supplementary Information 2.1: Whole genome sequencing of PI-7 transposon mutants). The PacBio genome sequences of all isolates has been deposited in Sequence Read Archive (SRA) repository within National Center for Biotechnology Information (NCBI) database under the Bioproject accession number PRJNA667599.

### Chlorination-Based Decay Kinetics

Chlorine was added to cell suspensions of PI-7 WT and mutants (*n* = 14) as diluted solution of NaClO. The NaClO stock (diluted 1000×) concentration was determined by DPD method. This method involves oxidation of an organic dye, N,*N* -Diethyl- *p*-phenylenediamine (DPD) with an oxidizing agent such as KMnO_4_ to produce an intensely colored Würster dye. The concentration of oxidizing agent is directly proportional to the amount of Würster dye produced, which is measured by recording the absorbance at 292 nm. Concentrations of KMnO_4_ is converted to equivalent Cl_2_ concentrations by applying the correction factor based on the difference in number of electrons donated by KMnO_4_ compared to Cl_2_ in accordance with the redox half-reactions. Relation between absorbance of Würster dye produced and concentration of oxidizing agent is defined by Beer’s law, as shown in equation below, where ε (ε = 362 L/mole/cm) is the molar absorptivity of the sample in L/mole/cm (calculated from the slope of calibration plot between known concentrations of KMnO4 and absorbance of Würster dye), L is the path length of the sample in cm and C is the concentration in moles/L (Johnson and Melbourne, 1996; Len et al., 2000; Xiong et al., 2017).

$$O\,D_{292}=ℰ\times \,L\times C$$

After determining the stock concentration, secondary NaClO stock concentrations of 100 mg/L were prepared. Overnight cultures of WT and mutants propagated in LB medium with 8 μg/mL meropenem were harvested at the late exponential phase, washed twice and then resuspended in 1× PBS (pH = 7.4) solution to OD_600_ of 1.0 (ca. 10^9^ CFU/mL). Homogeneous cell suspensions of WT and mutants were incubated with 3.25 mg/L Cl_2_, and were maintained in dark glass bottles at 37°C with shaking. Cell suspensions without addition of Cl_2_ were used as the control. Chlorination-based decay kinetics of WT and mutants were estimated based on cell counts of samples drawn between 0 and 80 min at intervals of 15 min during the first 60 min and then at intervals of 10 min and expressed in terms of half-lives (duration required to reduce the cell concentration by 50% upon chlorination) (Supplementary Table 1). Samples were quenched for chlorine through addition of excess Na_2_S_2_O_3_ (3-times in excess of Cl_2_ concentration). Cell counts were then measured in terms of colony forming units/mL (CFU/mL) plate counts by preparing serial dilutions (in 1× PBS, pH 7.5) of each temporal sample.

### UV Disinfection-Based Decay Kinetics

PI-7 WT and selected mutants (*n* = 14) were tested for UV disinfection-based decay kinetics. An 8W monochromatic UVP lamp emitting light at the germicidal UV-C (254 nm) wavelength (Analytik Jena, Upland, CA, United States) was used for conducting UV disinfection-based decay kinetics (Augsburger et al., 2019). Testing conditions were laid out, as described in a previous study (Augsburger et al., 2019). Overnight cultures of WT and mutants propagated in LB medium with 8 μg/mL meropenem were harvested at the late exponential phase, washed twice and then resuspended in 1× PBS (pH = 7.4) solution to OD_600_ of 1.0 (ca. 10^9^ CFU/mL). Homogenous cell suspensions (80 mL) of WT and mutants were pipetted to individual 100 mL glass beakers that were wrapped in black scotch tapes to prevent exposure to light from side walls of the beaker. Glass beakers were maintained on a magnetic stirrer to ensure continuous mixing. Temperature inside UV-C_254_ disinfection chamber was maintained at 37°C using a water bath. UV-based inactivation kinetics of WT and mutants in 1× PBS were estimated based on cell counts (CFU/mL) of samples drawn between 0 and 10 min at intervals of 2.5 min. CFU/mL was determined by plate counts after preparing serial dilutions (in 1× PBS) of each temporal sample. UV-based inactivation did not follow first order kinetics and hence, kinetics parameters such as half-lives were not determined. In addition, UV-based inactivation between PI-7 WT and mutants were expressed in terms of the time required to achieve a certain log reduction.

### Decay Kinetics Upon Solar Irradiation

PI-7 wild-type (WT) and selected mutants (*n* = 14) were tested for solar irradiation-based decay kinetics using a photosimulator carrying a xenon arc lamp (Atlas Suntest XLS + photosimulator, Chicago, IL, United States), as described previously (Al-Jassim et al., 2017). Briefly, overnight cultures of WT and mutants propagated in LB medium with 8 μg/mL meropenem were harvested at the late exponential phase, washed twice and then resuspended in 1× PBS (pH = 7.4) solution to OD_600_ of 1.0 (ca. 10^9^ CFU/mL). Homogenous cell suspensions (80 mL) of WT and mutants were pipetted to individual 100 mL glass beakers that were wrapped in black scotch tapes to prevent exposure to light from side walls of the beaker. Top of the glass beakers subjected to solar irradiation were covered with a glass filter (Newport Corporation, Irvine, CA, United States) that facilitates entry of light wavelengths ≥280 nm, while dark controls were covered with glass filters wrapped in aluminum foil. Exposure to solar irradiation was conducted by placing these beakers within the photosimulator, with the liquid content maintained at 37°C and homogenously mixed throughout the experiment. The inactivation curve of *E. coli* PI-7 in the same solar simulator was known from previous studies (Al-Jassim et al., 2017, 2018), where the bacterium exhibits no decay for the first 4 h [termed as lag phase (Supplementary Table 1)] before decaying. Aliquots of either WT or mutants were sampled from the individual beakers at regular intervals, namely 1 h during lag phase and 2 h during decay phase. Cell counts were measured in terms of colony forming units/mL (CFU/mL) plate counts by preparing serial dilutions (in 1× PBS) of each temporal sample. Solar inactivation curves were acquired by plotting temporal-based logarithmic cell density normalized with initial cell density (log_10_ scale) against time. Decay constant was derived from the slopes of solar inactivation curves plotted in natural log (ln) scale. Reduction in light penetration effected by the sample turbidity was mathematically corrected by applying a correction factor to the slope of decay curves, as described previously (Romero et al., 2011; Rice et al., 2012; Al-Jassim et al., 2017) prior to calculation of half-life and statistical estimations. Half-lives for WT and mutants (duration required to reduce the cell concentration by 50% upon solar irradiation) (Supplementary Table 1) were calculated by using corrected slopes of the decay kinetics curve in accordance with the first order decay kinetics equation, as outlined in our previous study (Al-Jassim et al., 2017). All tests described in disinfection sections were conducted in 3 biological trials, with each trial encompassing 3 technical replicates for every isolate.

### Statistical Estimations of Inactivation Curves

Statistical analysis of inactivation curves exhibited by PI-7 WT and mutants were conducted in accordance with the protocols mentioned in our previous study (Al-Jassim et al., 2017). Lag-phase lengths, corrected decay constants and half-lives of WT and mutants (determined after lag phase) conducted under different experimental conditions using 3 biological replicates, respectively, were compared by performing one-way ANOVA (2019 Microsoft Excel, Version 16.29). Single-regression analysis was used to compare each slope to 0 at α = 0.05 so as to determine if inactivation is observed. Acceptance of this hypothesis implied toward lack of inactivation and vice versa. Lag-phase is the initial phase in disinfection-inflicted decay kinetics, which reports lack of inactivation or minimal amount of decay (<1-log inactivation). In solar irradiation kinetics, lag-phase length was measured as the duration along which the hypothesis of slope to 0 at α = 0.05 was valid and hence supported minimal amount of bacterial inactivation upon exposure to solar irradiation (Al-Jassim et al., 2018).

## Results

### Phenotypic Traits Displayed by PI-7 Mutants Relative to WT

To identify the role of virulence traits in imparting enhanced tolerance to disinfection, we constructed *E. coli* PI-7 random transposon insertion site library through insertion of Tn5 transposon into *E. coli* PI-7 genome and selected a total of 14 mutants. The 14 isolates were grouped into 5 categories under the following considerations: (1) Implications of relative changes in virulence factors of each category upon its response to disinfection; (2) How transposon mutagenesis modulated the diverse spectrum of virulence factors harbored by *E. coli* PI-7. Based on these two considerations, the 5 categories of mutants are: Class A-Resistant isolates; Class B-Susceptible isolates; Class C- Isolates with enhanced virulence factors; Class D-Isolates with enhanced oxidative stress response; Class E-Isolates with enhanced motility/siderophore production. This style of classification broadens the understanding of how differential changes in virulence factors impact resistance of pathogens to disinfection.

**FIGURE 1:**
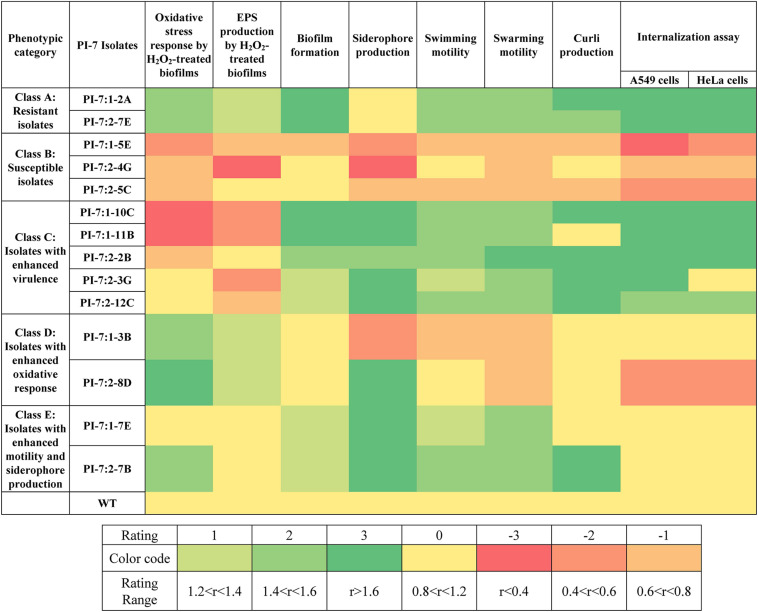
Phenotypic traits with respect to oxidative stress response and virulence traits displayed by selected *E. coli* PI-7 random transposon insertion site mutants relative to WT. Relative phenotypic traits higher than WT are shown in green. Rating of 3 indicates relative phenotypic traits >1.6-folds (*p* < 0.05), 2 indicates relative phenotypic trait between 1.4 and 1.6-folds (*p* < 0.05) and 1 indicates relative phenotypic traits between 1.2 and 1.4-folds (*p* < 0.05). Relative phenotypic traits lower than WT is shown in orange. Rating of -3 indicates relative phenotypic traits <0.4-folds (*p* < 0.05), –2 indicates relative phenotypic trait between 0.4 and 0.6-folds (*p* < 0.05) and -3 indicates relative phenotypic trait between 0.6 and 0.8-folds (*p* < 0.05). Relative phenotypic traits exhibiting no significant differences relative to WT are shown in yellow. Based on the phenotypic traits relative to WT, mutants are classified into five categories, namely class A (includes mutants with enhancement in nearly all examined phenotypic traits), class B (includes mutants with decrease in all examined phenotypic traits), class C (includes mutants with enhanced virulence traits), class D (includes mutants with enhanced oxidative stress response) and class E (includes mutants with enhanced motility and siderophore production).

Figure 1 illustrates the variation in oxidative stress response and virulence traits between PI-7 WT and mutants. A detailed elaboration on the differences in the phenotypic traits between mutants and *E. coli* PI-7 WT are described in Supplementary Information 3 (Section: Phenotypic Traits Of Mutants). In summary, Class A-Resistant isolates 1-2A and 2-7E displayed significantly higher levels of oxidative stress response (Figure 1 and Supplementary Figures 1, 2) and virulence traits such as biofilm formation, motility, curli production and internalization or adhesion into mammalian cells (Figure 1 and Supplementary Figures 3–7). Class B-Susceptible isolates 1-5E, 2-4G, and 2-5C exhibited lower oxidative stress response (Figure 1 and Supplementary Figures 1, 2) and virulence traits (Figure 1 and Supplementary Figures 3–7). Class C-Isolates with enhanced virulence traits 1-10C, 1-11B, 2-2B, 2-3G, and 2-12C exhibited significant enhancement in all virulence factors (Figure 1 and Supplementary Figures 3–7) coupled with either considerably decreased or relatively similar oxidative stress response compared to WT (Figure 1 and Supplementary Figures 1, 2). Class D-Isolates with enhanced oxidative stress response 1-3B and 2-8D exhibited significant increase in oxidative stress response (Figure 1 and Supplementary Figures 1, 2). Class E-Isolates with enhanced motility/siderophore were categorized primarily on the basis of their higher degree of siderophore production (Figure 1 and Supplementary Figure 4) and motility traits (Figure 1 and Supplementary Figure 5) relative to WT. One isolate, namely 2-7B, within Class E also has enhanced curli production (Figure 1 and Supplementary Figure 6).

**FIGURE 2:**
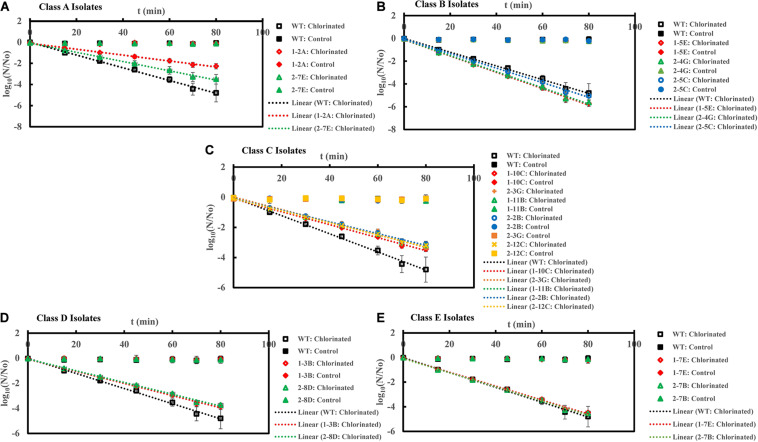
Inactivation curves of *E. coli* PI-7 WT and 5 categories of PI-7 transposon mutants exposed to chlorination in buffer. Best-fit curves for chlorination are reconstructed from the decay phase of inactivation curves. Half-lives derived from decay constants were also calculated from the portion corresponding to decay phase of chlorination in the inactivation curves. **(A)** Inactivation curves of PI-7 WT and resistant isolates (Class A isolates) exposed to chlorination; **(B)** Inactivation curves of PI-7 WT and susceptible isolates (Class B isolates) exposed to chlorination; **(C)** Inactivation curves of PI-7 WT and isolates with enhanced virulence (Class C isolates) exposed to chlorination; **(D)** Inactivation curves of PI-7 WT and isolates with enhanced oxidative stress response (Class D isolates) exposed to chlorination; **(E)** Inactivation curves of PI-7 WT and isolates with enhanced motility and siderophore production (Class E) exposed to chlorination.

### Genome Sequencing of Mutants

Based on our previous study, *E. coli* PI-7 harbors an extensive repertoire of virulence factors, which primarily contributed toward enhancing its persistence against solar irradiation (Al-Jassim et al., 2017). We used transposon mutagenesis specifically to construct a library of *E. coli* PI-7 mutants characterized with contrasting virulence traits, which could unveil how a suite of virulence factors facilitate the pathogens to survive against biocidal effects of disinfection. Transposon mutagenesis often disrupt the expression of genes adjacent to insertion sequences (Warnefors et al., 2010; Wang et al., 2013; Hutchison et al., 2019). Whole genome sequencing of shortlisted 14 mutants were conducted to identify the transposon mutagenesis-facilitated disruption sites in genes regulating virulence traits. Disruption of genes either regulating virulence traits or those directly associated with virulence factors could establish the connection between phenotype and genotype and hence, provide an understanding on how virulence factors harbored by PI-7 contribute toward enhancing the tolerance to disinfection. Detailed information of transposon insertions and deletions are shown in Supplementary Information 4 and Supplementary Tables 2–4. The presence and absence of transposon insertions and deletions in each mutant is illustrated in Supplementary Figure 8.

Majority of the transposon mutagenesis-based structural variants identified by whole genome sequencing seemed to be shared by all the shortlisted 14 mutants. Hence, this study has specifically considered only those genetically modified structural variants exhibited differently by each category of mutants compared to PI-7 WT. These structural variants have been previously reported to impact virulence factors at genetic level and hence, might play a role in regulating the phenotypic heterogeneity exhibited by each category of mutants. Although transposon mutagenesis disrupted ca. 3–4 genes associated with virulence, it did not adversely affect the growth of mutants (data not shown).

We further summarize the key genetic variants identified in each category of mutants. In case of resistant isolates (Class A), we specifically detected presence of transposon insertion sequence adjacent to potassium efflux system *kefA* (SVINS 82, Supplementary Figure 8A and Supplementary Table 2) a key component of the small mechanosensitive channel MscS in *E. coli*. In addition to its principal function as osmoregulators (Cui and Adler, 1996; Buda et al., 2016) in *E. coli*, mechanosensitive channels have been reported to have crucial links in regulating virulence factors; typically cell adhesion and biofilm formation (Cox et al., 2018). Class C isolates 1-11B and 2-2B also exhibited transposon insertion sites adjacent to potassium efflux system *kefA* (SVINS 82, Supplementary Figure 8A and Supplementary Table 2) and hence, displayed a profile in virulence traits similar to resistant isolates. However, these isolates also underwent deletion of regions flanking formate dehydrogenase (SVDEL 56, Supplementary Figure 8B and Supplementary Table 3), a key enzyme involved in mitigating oxidative stress damage in conjunction with glycine dehydrogenase (Alhasawi et al., 2015). In addition, we also detected transposon insertion between periplasmic phosphate binding protein PstS and ATP binding protein RbsA (SVINS 76, Supplementary Figure 8A and Supplementary Table 2), two significant components of ATP-binding family of transporters involved in virulence traits including biofilm formation (Zaborina et al., 2008), drug efflux (Ribič et al., 2020) and invasion (Jones, 2005) in Class C isolates (with the exception of 1-11B and 2-3G) and Class E isolate 2-7B. Class A, C, and E isolates shared two specific insertions SVINS 10 and 26, Supplementary Figure 8A and Supplementary Table 2) adjacent to genes reporting hypothetical functions. On the other hand, Class B susceptible isolates encountered deletion of regions between periplasmic phosphate binding protein PstS and ATP binding protein RbsA (either SVDEL 51, 52 or 53, Supplementary Figure 8B and Supplementary Table 4).

### Disinfection-Facilitated Inactivation Kinetics of PI-7 WT and Transposon Mutants

**FIGURE 3:**
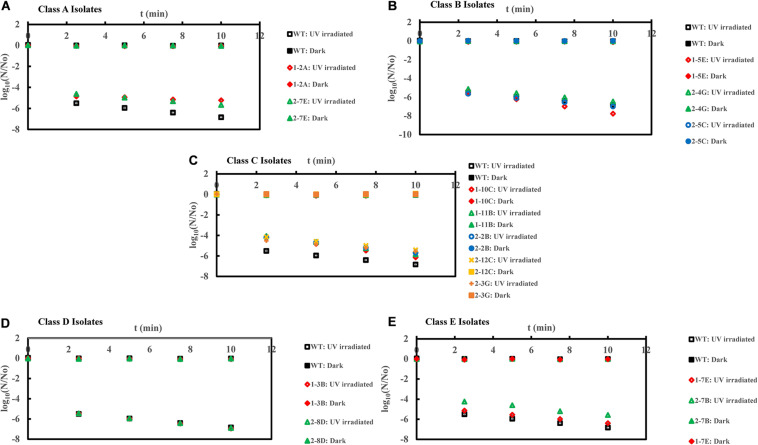
Inactivation curves of *E. coli* PI-7 WT and 5 categories of PI-7 transposon mutants exposed to UV irradiation in buffer. UV-based inactivation did not follow first order kinetics and hence, best-fit curves are not plotted as well as half-lives are not calculated. UV inactivation is expressed in terms of time required to achieve a certain log reduction. **(A)** Inactivation curves of PI-7 WT and resistant isolates (Class A isolates) exposed to UV; **(B)** Inactivation curves of PI-7 WT and susceptible isolates (Class B isolates) exposed to UV; **(C)** Inactivation curves of PI-7 WT and isolates with enhanced virulence (Class C isolates) exposed to UV **(D)** Inactivation curves of PI-7 WT and isolates with enhanced oxidative stress response (Class D isolates) exposed to UV; **(E)** Inactivation curves of PI-7 WT and isolates with enhanced motility and siderophore production (Class E) exposed to UV.

**FIGURE 4:**
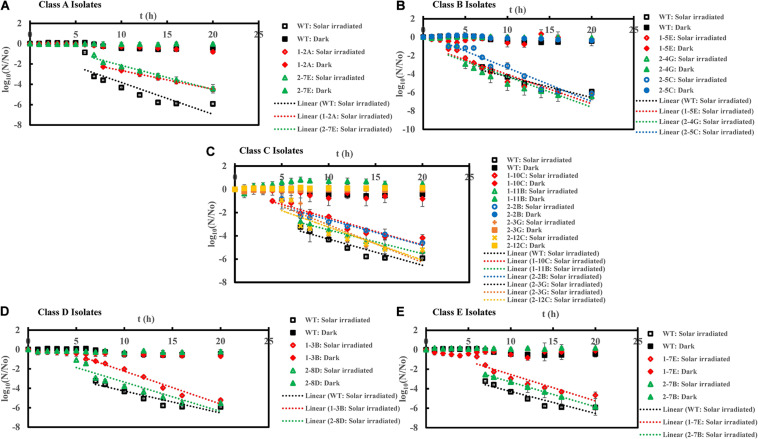
Inactivation curves of *E. coli* PI-7 WT and 5 categories of PI-7 transposon mutants exposed to simulated solar irradiation in buffer. Best-fit curves for simulated solar irradiation are reconstructed from the decay phase of inactivation curves. Half-lives derived from decay constants were also calculated from the portion corresponding to decay phase of solar irradiation in the inactivation curves. **(A)** Inactivation curves of PI-7 WT and resistant isolates (Class A isolates) exposed to simulated solar radiation; **(B)** Inactivation curves of PI-7 WT and susceptible isolates (Class B isolates) exposed to simulated solar radiation; **(C)** Inactivation curves of PI-7 WT and isolates with enhanced virulence (Class C isolates) exposed to simulated solar radiation; **(D)** Inactivation curves of PI-7 WT and isolates with enhanced oxidative stress response (Class D isolates) exposed to simulated solar radiation **(E)** Inactivation curves of PI-7 WT and isolates with enhanced motility and siderophore production (Class E) exposed to simulated solar irradiation.

To further investigate the role of virulence traits within PI-7 toward alleviating disinfection-imposed decay, we tested the decay kinetics of *E. coli* PI-7 WT and mutants upon exposure to 3 disinfection strategies, namely chlorination, UV and solar irradiation (Figures 2–4). We observed significantly longer half-lives in case of chlorination and solar irradiation for Class A-Resistant isolates 1-2A and 2-7E compared to WT coupled with enhanced tolerance following UV. Class B-Susceptible isolates 1-5E, 2-4G, and 2-5C exhibited shorter half-lives compared to WT when exposed to all three tested disinfection strategies. Class C-Isolates tend to have enhanced virulence traits (1-10C, 1-11B, 2-2B, 2-3G, and 2-12C) coupled with compromised oxidative stress response (Figure 1). There was an observed enhanced tolerance among these isolates to disinfection. Class D isolates 1-3B and 2-8D exhibited enhanced oxidative response but generally have lower virulence traits compared to WT. Class E-Isolates (1-7E and 2-7B) generally have higher biofilm formation, motility traits and siderophore production but lower oxidative stress response compared to WT.

### Inactivation Kinetics of PI-7 WT and Transposon Mutants Following Chlorination

Upon exposure to chlorination, class A resistant isolates 1-2A and 2-7E exhibited half-lives of 10.9 ± 1.7 min (*p* = 6.42 × 10^–10^) and 9.5 ± 1.6 min (*p* = 2.37 × 10^–8^), which was significantly longer than the half-life of WT (Half-life = 5.2 ± 0.2 min) (Figure 2A and Supplementary Table 1). When exposed to chlorine disinfection, class B susceptible isolates 1-5E, 2-4G, and 2-5C exhibited half-lives of 3.3 ± 0.1 min (*p* = 1.18 × 10^–11^), 3.3 ± 0.05 min (*p* = 1.32 × 10^–13^) and 3.6 ± 0.06 min (*p* = 1.09 × 10^–11^) compared to WT (Half-life = 5.2 ± 0.2 min) (Figure 2B and Supplementary Table 1). When treated with chlorine, class C isolates with enhanced virulence such as 1-10C, 1-11B, 2-2B, 2-3G, and 2-12C exhibited half-lives of 7.0 ± 0.08 min (*p* = 1.08 × 10^–11^), 7.6 ± 0.3 min (*p* = 1.06 × 10^–9^), 7.7 ± 0.2 min (*p* = 1.10 × 10^–10^), 7.2 ± 0.2 min (*p* = 1.09 × 10^–9^), and 7.5 ± 0.2 min (*p* = 1.72 × 10^–11^) (Figure 2C and Supplementary Table 1). Potential role of increased oxidative stress response in enhancing tolerance toward chlorination was observed in the case of class D isolates 1-3B (Half-life = 6.2 ± 0.14 min, *p* = 1.35 × 10^–6^) and 2-8D (Half-life = 6.8 ± 0.6 min, *p* = 2.34 × 10^–5^) (Figure 2D and Supplementary Table 1). Half-lives of class C and class D isolates were significantly longer than that observed for WT (Half-life = 5.2 ± 0.2 min) (Figure 2D). Upon chlorine exposure, half-lives of class E isolate 1-7E (Half-life = 5.3 ± 0.03 min, *p* = 0.18) and 2-7B (Half-life = 5.2 ± 0.05 min, *p* = 0.80) were found to be comparable with WT (Half-life = 5.2 ± 0.2 min) (Figure 2E and Supplementary Table 1).

### Inactivation Kinetics of PI-7 WT and Transposon Mutants Following UV

UV disinfection kinetics showed 5-log_10_ inactivation for class A resistant isolates 1-2A and 2-7E, respectively, after 10 and 7.5 min of UV exposure compared to WT (>6-log_10_ inactivation within 5 min of UV exposure (Figure 3A). Class B isolate 1-5E achieved ca. 7-log_10_ inactivation after 7.5 min of UV exposure, whereas UV-based decay kinetics of other class B isolates 1-4G and 2-5C was found to be comparable with WT (<7-log_10_ inactivation after 10 min of UV exposure) (Figure 3B). When exposed to UV, class C isolates achieved ca. 6-log10 inactivation after 10 min of UV exposure compared to WT (>6-log_10_ inactivation within 5 min of UV exposure) (Figure 3C). On the other hand, UV-based inactivation kinetics of class D isolates 1-3B and 2-8D were found to be comparable with that of WT (>6-log_10_ inactivation within 5 min of UV exposure (Figure 3D). Class E isolate 2-7B displayed enhanced tolerance to UV disinfection by achieving only ca. 5-log_10_ inactivation within 5 min of UV exposure compared to WT, which achieved >6-log_10_ inactivation within 5 min of UV exposure (Figure 3E). However, this trend was not reproducible, since isolate 1-7E displayed UV inactivation kinetics similar to that of WT (Figure 3E).

### Inactivation Kinetics of PI-7 WT and Transposon Mutants Following Solar Irradiation

Class A-Resistant 1-2A and 2-7E also showed high tolerance to solar irradiation, since they exhibited significantly longer lag phase duration [1-2A: 7.0 h (*p* = 5.54 × 10^–7^); 2-7E: 6.2 ± 0.1 h (*p* = 0.04)] and half-life duration [1-2A: 7.8 ± 0.4 min (*p* = 1.17 × 10^–10^); 2-7E: 7.2 ± 2.0 min (*p* = 1.79 × 10^–5^)] compared to WT (Lag phase = 5.6 ± 0.3 h; Half-life = 4.3 ± 0.1 min) (Figure 4A (Supplementary Table 1). Upon exposure to solar irradiation, class B isolates 1-5E exhibited a lag phase of 2.0 h (*p* = 2.20 × 10^–13^) and half-life of 2.2 ± 0.03 min (*p* = 2.57 × 10^–11^), while 2-4G exhibited lag phase of 3.3 ± 1.0 h (*p* = 1.14 × 10^–5^) and half-life of 3.6 ± 0.2 min (*p* = 4.0 × 10^–3^) (Figure 4B and Supplementary Table 1), significantly shorter than that of WT (Lag phase = 5.6 ± 0.3 h; Half-life = 4.3 ± 0.1 min). Although class B isolate 2-5C displayed a significantly shorter lag phase length [2.0 h (*p* = 2.20 × 10^–13^)], its half-life [4.2 ± 0.03 min (*p* = 0.71)] was not considerably different from that with WT (Half-life = 4.3 ± 0.1 min) upon solar irradiation (Figure 4B and Supplementary Table 1). When exposed to solar irradiation, Class C isolates exhibited shorter or similar lag phases but considerably longer half-life [1-10C (Lag phase = 3.7 ± 0.2 h, *p* = 2.56 × 10^–7^; Half-life = 7.9 ± 0.1 min, *p* = 9.15 × 10^–14^), 1-11B (Lag phase = 5.3 ± 0.2 h, *p* = 0.176; Half-life = 7.2 ± 2.6 min, *p* = 1.0 × 10^–3^), 2-2B (Lag phase = 6.0 h, *p* = 0.06; Half-life = 7.3 ± 1.7 min, *p* = 5.3 × 10^–6^), 2-3G (Lag phase = 5.0 h, *p* = 1.0 × 10^–3^; Half-life = 7.4 ± 1.8 min, *p* = 8.51 × 10^–5^), and 2-12C (Lag phase = 3.7 ± 0.1 h, *p* = 6.29 × 10^–5^; Half-life = 7.4 ± 0.6 min, *p* = 1.14 × 10^–8^)] (Figure 4C and Supplementary Table 1) compared to WT (Lag phase = 5.6 ± 0.3 h; Half-life = 4.3 ± 0.1 min). Solar inactivation kinetics revealed no significant difference in lag phase of 6.2 ± 0.7 h (*p* = 0.10) and half-life of 3.7 ± 0.3 min (*p* = 0.08) for class D isolate 1-3B compared to WT (Figure 4D and Supplementary Table 1). A contradictory response was, however, observed for the other class D isolate 2-8D which has a shorter lag phase of 4.2 ± 0.2 h (*p* = 3.51 × 10^–5^) but no significant difference in half-life of 4.2 ± 0.04 min (*p* = 0.59) compared to WT (Lag phase = 5.6 ± 0.3 h; Half-life = 4.3 ± 0.1 min) (Figure 4D and Supplementary Table 1). Upon exposure to solar irradiation, class E isolates 1-7E and 2-7B displayed shorter lag phases (1-7E: Lag phase = 5.1 ± 0.1 h, *p* = 0.01; 2-7B: Lag phase = 4.7 ± 0.2 h, *p* = 0.17) but considerably longer half-lives (1-7E: Half-life = 12.03 ± 0.5 min, *p* = 3.38 × 10^–15^; 2-7B: Half-life = 8.02 ± 1.6 min, *p* = 2.03 × 10^–7^) compared to WT (Lag phase = 5.6 ± 0.3 h; Half-life = 4.3 ± 0.1 min) (Figure 4E and Supplementary Table 1).

## Discussion

In our previous study, we demonstrated the ability of opportunistic pathogen *E. coli* PI-7 to survive solar irradiation-imposed inactivation more effectively than commercial strain *E. coli* DSM1103 (Al-Jassim et al., 2017). Specifically, *E. coli* PI-7 exhibited longer half-life and lag phase than DSM 1103 prior to undergoing >5-log_10_ decay (Al-Jassim et al., 2017). Transcriptomic analysis confirmed the upregulation of genes associated with virulence factors, cellular repair and oxidative stress response that facilitated enhanced survival under solar radiation but did not confirm whether virulence factors were indeed playing a role in facilitating persistence (Al-Jassim et al., 2017).

Virulence profile of each category of mutants corroborated well with their respective disinfection-inflicted inactivation kinetics. Class A resistant isolates exhibited significantly higher oxidative stress response and virulence factors, which contributed to their higher persistence toward all deployed disinfection strategies. Similarly, enhanced virulence traits harbored by class C isolates contributed to longer persistence against chlorination, UV and solar irradiation. Higher oxidative stress response facilitated class D isolates to persist longer in disinfection processes involving oxidative damage such as chlorination but not to UV disinfection, while class E isolates with enhanced siderophore production and motility traits exhibited relatively similar responses as control WT upon exposure to diverse disinfection strategies. Class B isolates, which displayed repressed virulence factors and oxidative stress response, were found to be most susceptible against chlorination, UV (with exception of 2-5C) and solar irradiation compared to the other classes.

One of the virulence factors prevalent in Class A isolates is EPS production and biofilm formation, both of which form the primary requisites to inflict clinical infections as well as the first line of defense against external stressors (Chua et al., 2015). Virulent strains have the tendency to form biofilms upon exposure to stress conditions (Solano et al., 1998; Bonafonte et al., 2000; Al Safadi et al., 2012). EPS reduces the mass transfer of external stressors such as disinfectants and biocides (Stewart, 1996, 2003) as well as serve as a barrier against solar and UV irradiation (Ehling-Schulz et al., 1997; Kehr and Dittmann, 2015) and hence, reduces the effectiveness of disinfection strategies. Increase in biofilm formation and EPS production were further demonstrated to play a key role in lengthening the half-life and lag phase length of resistant (Class A) upon exposure to disinfection (Supplementary Figures 1–3).

Increase in biofilm formation often associates with a wide range of multidrug efflux pump and transporters (Upadya et al., 2011), which comprises of specialized membrane-associated proteins. For example, Class A and few Class C (1-11B and 2-2B) isolates achieved increased persistence to disinfection likely due to induction of KefA. KefA is involved with providing sensitivity to K^+^ concentration and also play a role in regulating the activity of small mechanosensitive channels (Cui and Adler, 1996) that govern osmoregulation in *E. coli* (Cui and Adler, 1996; Buda et al., 2016). In addition, previous studies have linked mechanosensitive channels with virulence of *Pseudomonas aeruginosa* related to mammalian pathogenicity (Tan et al., 1999) and they also have been reported to play a certain undefined role in biofilm formation (Cox et al., 2018). Through osmoregulation, multidrug efflux pumps remove intracellular biocides to concentration below threshold level, hence paving the way for cellular detoxification (Upadya et al., 2011; Blanco et al., 2016). In our earlier study, cellular detoxification facilitated by upregulated multidrug efflux pumps and transporters played a key role in enhanced response of *E. coli* PI-7 toward solar inactivation (Al-Jassim et al., 2017). The detoxification role of multidrug efflux pumps and transporters among isolates exposed to chlorination have also been reported previously (Chang et al., 2007; Blanco et al., 2016).

Furthermore, inactivation response of isolates with enhanced virulence traits (1-10C, 1-11B, 2-2B, 2-3G, and 2-12C) also confirmed the role of oxidative stress response harbored by *E. coli* PI-7 in enhancing its tolerance to chlorination and solar irradiation (Figure 4). For example, NADPH-dependent glycine dehydrogenase mediates the hydrolysis of glycine to glyoxylate that scavenges H_2_O_2_ to generate formate (Alhasawi et al., 2015; Thomas et al., 2016). NADP^+^-dependent formate dehydrogenase oxidize formate with recirculation of NADPH to sustain H_2_O_2_ scavenging by glycine dehydrogenase. Hence, genetic disruption of formate dehydrogenase might result in suppression of oxidative damage control induced by sunlight and chlorine but not UV, as observed in the case of isolates 1-10C, 1-11B, 2-2B, and 2-12C.

Impact of biofilm formation, oxidative stress and virulence factors on disinfection is validated by Class B susceptible isolates. Biofilm formation and EPS production of all susceptible isolates was lower than WT and resulted in faster inactivation kinetics. In addition, the three isolates within Class B had a deletion in the pst-gene network (SV-Del 51, 52 or 53). Deletion of *pst*-gene network significantly suppressed virulence of *Mycobacterium smegmatis* (Banerjee et al., 2000), and that deletion of Pst system in uropathogenic *E. coli* led to attenuation of virulence and repression in the expression of type-1 fimbriae (Crépin et al., 2017). It can be suggested that the Tn5-modulated deletion of regions adjacent to PstS could have attenuated virulence factors of susceptible isolates. Therefore, lower tolerance of Class B susceptible isolates to the chlorine and solar irradiation could be attributed to their significantly decreased oxidative stress response and virulence factors (Figure 3).

Likewise, motility traits are crucial parameters that impact biofilm architecture of *E. coli* isolates (Yang et al., 2018). High expression levels of motility traits facilitates formation of vertical biofilm architecture with greater biofilm thickness (Wood et al., 2006), which disrupts the mass transfer of biocides as well as block the penetration of solar and UV irradiation (Bridier et al., 2011), as is observed for most of class A and C isolates with enhanced motility traits (Figure 1). However, Class E isolates, which had enhanced motility and siderophore production, exhibit no significant differences in their inactivation half-lives compared to control WT. These observations suggest that siderophore production likely does not contribute to enhance survival against disinfection strategies.

Enhanced curli production engineers stable biofilm formation (White et al., 2006) and also offers protection against stressors such as Cl_2_ (White et al., 2006; Wang et al., 2012). Curli expression has been reported to be directly correlated with enhanced biofilm formation (Wang et al., 2012), as observed in the case of resistant isolates, isolates with enhanced virulence traits as well as 2-7B (Supplementary Figure 6). Hence, it can be suggested that enhanced curli production together with potential induction of motility and other virulence factors through Tn5 insertion between periplasmic phosphate binding protein PstS and ATP binding protein RbsA (STINS 76, Supplementary Figure 8A and Supplementary Table 2) contributed toward lengthening the half-life of 2-7B upon exposure to solar irradiation (Supplementary Figure 6). Increased curli production also leads to enhanced bacterial cell internalization, as is evident from the high degree of mammalian cells internalization rate exhibited by resistant and virulent isolates (Supplementary Figure 7).

The findings of this study are in alignment with an earlier study showing the survival of uropathogenic *E. coli* strains in sewage treatment plants deploying chlorination and UV irradiation (Anastasi et al., 2013). Similarly, enhanced virulence facilitates the survival of pathogens like *Legionella pneumophila* in eukaryotic protozoan cell types such as *Acanthamoeba castellanii*, which provides robust environment for pathogens to survive and offer protection against biocides and external stressors such as Cl_2_ and UV (Cirillo et al., 1999).

This study further demonstrated a suite of virulence traits (i.e., biofilm formation, EPS production, motility, curli production, and internalization capability) play a role in enhancing the survival of pathogens against disinfection. However, absence of quantitative validation of expression changes in virulence genes modulated by transposon mutagenesis might be a limiting factor to address the role of virulence traits in abating disinfection at molecular level. Further studies at transcriptomic level monitoring the expression changes in virulence genes harbored by *E. coli* PI-7 random transposon mutants could elucidate the crucial role of virulence traits in *E. coli* PI-7 toward attenuating the disinfection-imposed inactivation.

Although not within the scope of present study, this study could be expanded to extrapolate and isolate specific determinants of virulence as well as the wider arsenal of alternative protective mechanisms harbored by *E. coli* PI-7 such as cellular repair and oxidative stress response (Al-Jassim et al., 2017) through site-directed mutagenesis from a single genetic change and determine how those changes (and the specific trait) influence resistance to disinfectants. Alternatively, a parallel study conducted on deletion or complementation of genes that is established as key modulators of virulence factors (e.g., *kef*A or *pst*S or oxidative stress response inducing NADP^+^-dependent formate dehydrogenase) in pathogenic *E. coli* PI-7 could ascertain the impacts of transposon mutagenesis-based genetic variants on observed phenotypic heterogeneity in PI-7 mutants relative to WT. Availability of this data could also possibly rule out the potential polar effects of transposon insertions.

Despite these study limitations, through the screening and selection of mutants from the random transposon mutagenesis library of *E. coli* PI-7 primarily on the basis of biofilm formation capability, we have demonstrated that certain virulence factors assist pathogens to exhibit longer persistence to oxidation-based disinfection strategies (e.g., chlorine and solar irradiation) but such traits are relatively less useful to survive against UV. Coupled with earlier studies that suggest the use of UV does not contribute to horizontal gene transfer of genes (Augsburger et al., 2019) unlike other disinfection strategies (Zhang et al., 2017), our findings reiterate that UV could contribute to a more effective inactivation of virulent bacteria in water matrices of high transmissivity. Alternatively, given that strains with enhanced biofilm capabilities and associated virulence traits tend to persist longer in chlorine, relatively longer contact time can be used to improve disinfection efficacy. A longer contact time can also be coupled with the use of monochloramine, which was shown to have better penetration abilities than chlorine through biofilm (Lee et al., 2018) to disinfect treated wastewater.

## Data Availability Statement

The original contributions presented in the study are publicly available. This data can be found here: https://www.ncbi.nlm.nih.gov/bioproject/PRJNA667599.

## Author Contributions

KS designed and performed the experiments, data analysis, and wrote the manuscript. RL performed the analysis of whole genome sequencing data and wrote the manuscript. AR developed the protocol for conducting mammalian cell internalization assay. NA provided advice for conducting UV and solar irradiation tests. NZ developed the protocol for chlorination tests. AR, NA, and NZ also provided comments on improving the manuscript. JT contributed to analysis of whole genome sequencing data and provided comments on improving the manuscript. P-YH conceived and designed the experiments, analysis and interpretation of data, wrote the manuscript, supervised the research, and provided reagents and materials. All authors contributed to the article and approved the submitted version.

## Conflict of Interest

The authors declare that the research was conducted in the absence of any commercial or financial relationships that could be construed as a potential conflict of interest.

## Supplementary Material

Supplementary Figure 1 Response of PI-7 WT and mutant biofilms exposed to oxidative stress induced by exposure to H_2_O_2_ for 1h, Effect of H_2_O_2_-induced oxidative stress on EPS production within biofilms of PI-7 WT and mutants, Biofilm formation exhibited by PI-7 WT and mutants, Siderophores produced by PI-7 WT and mutants, Swimming and Swarming motility exhibited by PI-7 WT and mutants, Relative amount of curli fibers released into cell exterior by PI-7 WT and mutants, Internalization of PI-7 WT and mutants onto mammalian cells, Genotype matrix representing transposon insertions and deletions in *E. coli* PI-7 random transposon mutants.

Supplementary Table 1 Disinfection kinetics parameters of *E. coli* PI-7 WT and different categories of mutants upon exposure to chlorination and solar irradiation.

Supplementary Table 2 List of Tn5 insertions in *E. coli* PI-7 transposon mutants with details of genes upstream and downstream of insertion locus, List of genes overlapping with Tn5-mediated deletions in *E. coli* PI-7 transposon mutants, List of genes flanking Tn5-mediated deletions in *E. coli* PI-7 transposon mutants.

Supplementary Methods 1 Phenotypic traits-based characterization of PI-7 mutants, Phenotypic traits of PI-7 mutants, Whole Genome Sequencing of PI-7 transposon mutants, Genomic analysis of isolates from *E. coli* PI-7 random transposon insertion site library.

Supplementary Results 1 Oxidative stress response of PI-7 WT and mutants, Virulence factors exhibited by PI-7 WT and mutants.
