# Supplementary material for: Elucidating the Role of Virulence Traits in the Survival of Pathogenic E. coli PI-7 Following Disinfection
Source: Front Bioeng Biotechnol. 2020 Dec 22;8:614186. doi: 10.3389/fbioe.2020.614186 (PMC7783314; doi:10.3389/fbioe.2020.614186)
Supplement: Supplementary Figure 1 — Response of PI-7 WT and mutant biofilms exposed to oxidative stress induced by exposure to H2O2 for 1h, Effect of H2O2-induced oxidative stress on EPS production within biofilms of PI-7 WT and mutants, Biofilm formation exhibited by PI-7 WT and mutants, Siderophores produced by PI-7 WT and mutants, Swimming and Swarming motility exhibited by PI-7 WT and mutants, Relative amount of curli fibers released into cell exterior by PI-7 WT and mutants, Internalization of PI-7 WT and mutants onto mammalian cells, Genotype matrix representing transposon insertions and deletions in E. coli PI-7 random transposon mutants. [file Data_Sheet_1.pdf]

Supplementary Information

Elucidating the role of virulence traits in the survival of pathogenic *E. coli* PI-7 following disinfection

Krishnakumar Sivakumar[1], Robert Lehmann[2], Andri Taruna Rachmadi[3], Nicolas Augsburger[3], Noor Zaouri[3], Jesper Tegner[2], Pei-Ying Hong[3*]

1. King Abdullah University of Science and Technology, Computational Bioscience Research Center, Biological and Environmental Science and Engineering Division, Thuwal, Saudi Arabia
2. King Abdullah University of Science and Technology, Living Systems Laboratory, Environmental Epigenetic Program, Biological and Environmental Science and Engineering Division, Computer, Electrical and Mathematical Sciences and Engineering Division, King Abdullah University of Science and Technology (KAUST), Thuwal, 23955–6900, Saudi Arabia.
3. King Abdullah University of Science and Technology, Water Desalination and Reuse Center, Biological and Environmental Science and Engineering Division, Thuwal, Saudi Arabia

*** Corresponding author**

Email: peiying.hong@kaust.edu.sa

Telephone: +966 12 808 221

1. PHENOTYPIC TRAITS-BASED CHARACTERIZATION OF PI-7 MUTANTS

SI-1.1: Invasiveness assay of PI-7 WT and mutants on mammalian cells

Invasiveness assay using PI-7 WT and mutants (n = 14) were performed on HeLa and human alveolar epithelial A549 cells in accordance with our previous study (Mantilla-Calderon et al., 2016). Both human cell-lines were procured from ATCC (CCL-2). HeLa cells were propagated in Dulbecco's Modified Eagle's medium (DMEM) (Invitrogen, San Diego, CA, USA) with 10 % fetal bovine serum (FBS) (Thermo Fisher Scientific, Carlsbad, CA, USA) supplemented with 1% penicillin/streptomycin (Thermo Fisher Scientific, Carlsbad, CA, USA)(Mantilla-Calderon et al., 2016). A549 cells were grown in Ham's F12 medium with 10% FBS supplemented with 1% penicillin/streptomycin and 14 mM $NaHCO_3$ (Rubens et al., 1992;Gaddy et al., 2009). Cells were incubated in 75 cm^2 vent-cap sterile non-pyrogenic polystyrene tissue culture flasks (VWR, Radnor, PA, US) at 37 ^{O}C with 5 % CO_2. After attaining ~90% confluence, 1 mL cells were seeded into each wells of a 24-well clear tissue culture-treated plate (Costar, Corning Inc, NY, USA) containing either DMEM with 10 % FBS supplemented with 1% penicillin/streptomycin (for HeLa cells) or Ham's F12 medium with 10% FBS supplemented with 1% penicillin/streptomycin and 14 mM $NaHCO_3$ (for A549 cells). Mammalian cell cultures were incubated overnight in 37 °C, 5% CO_2. In addition to preparing the mammalian cell cultures for invasive tests, PI-7 WT and mutants were also grown from a single colony into LB medium with 8 µg/mL meropenem and incubated under aerobic conditions at 37 °C until a cell density equivalent to OD_{600} of 0.8 was achieved. Bacterial cells were then centrifuged at 5000 *g* for10 min, harvested and resuspended in DMEM and F-12 medium respectively. About 300 µL bacterial suspension were then added to HeLa cells and A549 cells, respectively. After inoculation of bacterial cells, HeLa and A549 cell-lines were incubated for 2 h at 37 °C in presence of 5 % CO_2 followed with the addition of 1 mg/mL lysozyme

(Sigma-Aldrich, St. Louis, MO, USA). After 4 h of incubation, cells were initially washed twice with PBS (1x) and then treated with 0.2% Triton in PBS (1x) for 20 min at 4 ºC. Treatment with 0.2% Triton breaks open mammalian cells and facilitates the release of bacterial cells into supernatant, which is collected through centrifugation (1000 x g, 4 min) (Al-Jassim et al., 2017). 100 μL of supernatant were then plated on LB agar plates supplemented with 8 μg/mL meropenem to quantify the amount of bacterial cells internalized within the mammalian cells. Invasiveness of PI-7 WT and mutants on HeLa and A549 cells were quantified in terms of bacterial internalization rate

$$\text{Bacterial internalization rate} = \frac{\dfrac{\text{Bacterial cell density internalized on mammalian cells}}{\text{Bacterial cell density inoculated on mammalian cells}}}{\text{Internalization period}}$$

A total of 4 biological replicates (with 3 technical replicates per biological replicate) per isolate of PI-7 or mutants was conducted for this test.

SI-1.2: Motility-associated traits

Motility is a virulence factor that directs the bacteria to advance along chemical gradients. Flagellar-mediated motility regulates cell adherence and hence, play a central role during initial phases of bacterial infection (Josenhans and Suerbaum, 2002;Haiko and Westerlund-Wikström, 2013). PI-7 WT and mutants were monitored for flagellar-mediated motility traits such as swimming and swarming. Swimming motility was evaluated by spotting 1.5 μL PI-7 WT and mutants at the center of semi-solid agar medium composed of 5 g/L peptone, 3 g/L yeast extract and 3 g/L Difco-Bacto agar (Difco Laboratories Inc, Detroit, MI, USA). Plates were incubated at 37 °C for 24 h and the plates dried for 9 h. Extent of swimming motility was evaluated in terms of diameter of swimming zone within the agar medium (Chow et al., 2011;Armbruster et al., 2013).

Swimming zone was considered to be an ellipse and its diameter along the major and minor axis was resolved using ImageJ (Version 1.46r, National Institute of Health, Bethesda, MD, USA).

To assess swarming motility, 1.5 μL of PI-7 WT and mutants were pipetted on the surface of agar composed of 5 g/L tryptone, 2.5 g/L yeast extract, and 10 g/L Difco-Bacto agar. After incubation at 37 °C for 48 h, the diameter of swarming motility (migration of the bacteria through the agar) from the center towards the periphery of the plate was resolved using ImageJ. Diameter of swimming and swarming zone were resolved using ImageJ in pixels and then converted to metric scale using diameter of the petri dish as reference. A total of 6 biological replicates were conducted for swimming and swarming assays, respectively.

SI-1.3: Curli production assay by PI-7 WT and mutants

E. coli produces extracellular appendages such as curli fibers, which contributes to cell-cell interactions and cell adhesion. Curli exhibits characteristics of amyloid fibers and has been reported to facilitate cell aggregation during urinary tract infection by uropathogenic *E. coli* (Chapman et al., 2002) as well as cell adherence of avian pathogenic *E. coli* (Gophna et al., 2001) and internalization of HeLa cells (Gophna et al., 2001). Most of these reported studies contribute to consider curli as a virulence factor (Saldaña et al., 2009). A congo red depletion assay was performed to evaluate and compare the extent of curli production among PI-7 WT and mutants (Saldaña et al., 2009;Carter et al., 2016). Overnight LB (with 8 μg/mL meropenem) grown cultures of PI-7 WT and mutants were harvested and resuspended in 0.9% NaCl at varying cell densities equivalent to OD_{600} of 0.25, 0.5, 0.75 and 1.0. Whole-cells of PI-7 WT and mutants were treated with 10 μg/mL of congo red. After 10 min of incubation under shaking conditions, cells were centrifuged (8000 x g, 10 min) and cell-free supernatant was collected after discarding the pellet. Amount of congo red released into the supernatant was measured in terms of OD_{490} normalized

with cell density (OD_{600}). Congo red strongly binds with curli fibers and hence, decreased amount of congo red in supernatant is a relative measure of enhanced curli production. Two independent biological trials were conducted for congo red-based curli production assay with 3 technical replicates per biological replicate).

SI-1.4: Biofilm formation by PI-7 WT and mutants

Ability to colonize and form biofilms on host cells by pathogenic bacteria often serve as the significant virulence factor towards chronic pathogenic infections (O'Loughlin et al., 2013;Stępień-Pyśniak et al., 2019). Initial screening and selection of mutants were conducted by monitoring the biofilm formation capability of mutants from the PI-7 transposon library relative to WT using static biofilm assay (Sivakumar et al., 2015;Sivakumar et al., 2019). Briefly, biofilms of WT and mutants were propagated in M9 medium with 1/10th LB (10% by volume) at 37°C on a polystyrene flat bottom 96-well plate (Costar, Corning Inc., Corning, NY, United States). M9 media was composed of 3 g/L KH_2PO_4, 12.8 g/L Na_2HPO_4, 0.5 g/L NaCl, 1 g/L NH_4Cl, 0.24 g/L $MgSO_4$, 11.1 mg/L $CaCl_2$. 10% LB was used as the sole carbon source in M9 medium. Biofilm formation capability of PI-7 WT and mutants was monitored for 48 h at equal intervals of 6 h in separate microplate biofilm reactors (96-well plate). After stipulated time interval, planktonic cells from each respective biofilm reactors were discarded and those adhered to base of the wells were washed with 0.9% NaCl. Biofilm-based cells were then subjected to crystal violet (CV) staining (100 µL, 1% v/v) followed with incubation for 15 min at room temperature. After removal of excess CV, well-adhered cells were re-suspended in ethanol and the biofilm-based biomass was recorded at OD_{590} using a microplate reader (SpectraMax 340PC384, Molecular Devices, CA, United States). Experiment was conducted using 4 biological replicates with 3 technical replicates per biological replicate.

SI-1.5: Siderophore production by PI-7 WT and mutants

Siderophores contribute to bacterial virulence by compromising the iron-withholding defense systems of host cells (Ramanan and Wang, 2000;Lamont et al., 2002). Siderophores are low molecular weight iron-chelators, which mediates the translocation of iron ions from extracellular environment into bacterial cells through specific cell surface receptors (Ramanan and Wang, 2000). PI-7 WT and mutants were tested for siderophore production by conducting $FeCl_3$ assay (Neilands, 1981;Neilands and Nakamura, 2017). PI-7 WT and mutants were grown in iron-deficient M9 and M63 media with 30 mM glucose. M63 medium was composed of 2 g/L $(NH_4)_2SO_4$, 13.6 g/L KH_2PO_4, 1 g/L Casamino acids, 0.0005 g/L thiamine and 0.24 g/L $MgSO_4$. Filtered cell free supernatants of PI-7 WT and mutants grown in M9 and M63 media were mixed with 2.5% $FeCl_3$ at a volumetric proportion of 1:5. Amount of siderophore generated were recorded at OD_{490} using a microplate reader (SpectraMax 340PC384, Molecular Devices, CA, United States). Initially, an absorption spectrum between 350-700 nm at 5 nm wavelength intervals prepared for WT showed its absorption maxima at 490 nm and hence, quantification of siderophore generated was recorded at OD_{490}. Study was conducted using 4 biological replicates with 3 technical replicates per biological replicate.

SI-1.6: Oxidative stress response by biofilms of PI-7 WT and mutants

Our previous study had demonstrated the role of oxidative stress response in PI-7 towards mitigating against reactive oxygen species (ROS)-based stress induced by solar irradiation (Al-Jassim et al., 2017). Mutants were screened by monitoring their response to oxidative stress relative to WT. Biofilms of WT and mutants were propagated for 24 h in M9 medium with $1/10^{th}$ LB (10% by volume) at 37 ºC on a polystyrene flat bottom 96-well plate. After 24 h, biofilms were treated with M9 medium supplemented with 12.5 mM H_2O_2 for 1 h. Biofilm response to H_2O_2-

induced oxidative stress was monitored by quantifying the well bottom-adhered cell density after H_2O_2 treatment by conducting static biofilm assay as described in the biofilm formation section. Study was conducted using 4 biological replicates with 3 technical replicates per each biological replicate. EPS produced by WT and mutants were quantified through Duboi's assay (Taylor, 1995). Spent medium was removed from the wells and used for EPS quantification after harvesting planktonic cells through centrifugation. Briefly, 20 μL of cell-free supernatant was mixed with 20 μL of phenol (4% w/v) and 160 μL of H_2SO_4 (98%) and incubated at room temperature for 10-15 min. The OD_{490} of the reaction mixture was measured to quantify the EPS produced by *E. coli* PI-7 WT and mutants. To quantify the relative measure of oxidative stress response by H_2O_2-treated mutant biofilms, biofilm biomass and EPS production exhibited by each mutant was normalized against that of WT.

Biofilms of WT and mutants were also cultivated in M9 medium with $1/10^{th}$ LB on cellulose acetate (CA) membrane coupons (5 mm x 5 mm) arranged inside each well of a 24-well plate. After 72 h, CA coupon-adhered biofilms were treated with 12.5 mM H_2O_2 for 1 h. Biofilm response to H_2O_2-induced oxidative stress was examined by quantifying the cell detachment rate defined as the amount of cells detached from the coupons (measured as CFU/mL from the 0.9% NaCl cell suspension) per coupon surface area per unit time ($CFU/mm^2/min$). Cell detachment rate is used as a supplementary data to support biofilm biomass in evaluating oxidative stress response by biofilms of WT and mutants.

2. GENOMIC CHARACTERIZATION OF PI-7 TRANSPOSON MUTANTS

SI-2.1: Whole Genome Sequencing of PI-7 Transposon Mutants

Genomic DNA was extracted from WT PI-7 and 14 mutants using a DNeasy blood and tissue kit (Qiagen, Hilden, Germany) (Mantilla-Calderon et al., 2016) and the whole genome sequencing was conducted using the PacBio Sequel platform. The read quality was evaluated using FastQC before mapping to the previously published reference genome assembly of *E. coli* PI-7 (Mantilla-Calderon et al., 2016) using minimap2 (Li., 2018) via the PacBio wrapper pbmm2 version 1.1.0 (https://github.com/PacificBiosciences/pbmm2/). Structural variants among the isolates were then detected using pbsv 2.2.2 (https://github.com/PacificBiosciences/pbsv). Similarity to known transposon sequences was tested using ISfinder (Siguier et al., 2006). The PacBio genome sequences of all isolates has been deposited in Sequence Read Archive (SRA) repository within National Center for Biotechnology Information (NCBI) database under the Bioproject accession number PRJNA667599.

3. PHENOTYPIC TRAITS OF MUTANTS

SI-3.1: Oxidative response by biofilms of PI-7 WT and mutants

Enhanced oxidative stress response by *E. coli* PI-7 contributed to its longer persistence compared to commensal strain *E. coli* DSM1103, when exposed to solar irradiation. Our previous study also reported on the upregulation of genes associated with H_2O_2 response such as catalase and cytochrome c peroxidase in response to solar inactivation, which confirmed H_2O_2 to the predominant ROS stress inducer (Al-Jassim et al., 2017). To further confirm the extent of oxidative stress response in presence of H_2O_2 radicals, biofilms of WT and mutants grown for 24 h were treated with H_2O_2 and their oxidative stress response were evaluated in terms of biofilm biomass,

cell detachment rate and EPS production upon H_2O_2 treatment. Supplemental Figures S1A, S1B and S2 illustrated the biofilm biomass, cell detachment rate and EPS production by PI-7 WT and mutant biofilms upon induction of oxidative stress with H_2O_2 treatment.

SI-3.2: Oxidative response by biofilms of resistant isolates (Class A)

Resistant isolates exhibited significantly higher biofilm retention capability and produced more EPS than WT in presence of H_2O_2. Upon H_2O_2 treatment, resistant isolates 1-2A ($OD_{590} = 0.68 \pm 0.12$) and 2-7E ($OD_{590} = 1.12 \pm 0.15$) retained 1.5-times ($p = 3.10 \times 10^{-4}$) and 2.5-times ($p = 6.10 \times 10^{-7}$) higher biofilm biomass than WT ($OD_{590} = 0.45 \pm 0.07$) (Figure 1, Supplemental Figure S1A). Lower rates of cell detachment rates were observed for 1-2A ($6.5 \times 10^7 \pm 10.0 \times 10^5$ CFU/mm^2/min, $p = 9.0 \times 10^{-31}$) and 2-7E ($7.40 \times 10^7 \pm 1.20 \times 10^6$ CFU/mm^2/min, $p = 4.20 \times 10^6$) biofilms exposed to H_2O_2 treatment compared to WT ($1.30 \times 10^8 \pm 1.0 \times 10^6$ CFU/mm^2/min) (Figure 1, Supplemental Figure S1B). Additionally, 1-2A and 2-7E biofilms produced 1.3-times ($p = 1.7 \times 10^{-3}$) and 1.4-times ($p = 4.5 \times 10^{-6}$) higher EPS than WT in the presence of H_2O_2 (Figure 1, Supplemental Figure S2).

SI-3.3: Oxidative response by biofilms of susceptible isolates (Class B)

The presence of H_2O_2 significantly affected the biofilms of susceptible isolate 1-5E, as it could retain only 0.6-times ($OD_{590} = 0.27 \pm 0.04$, $p = 4 \times 10^{-3}$) lower biofilm biomass than WT ($OD_{590} = 0.45 \pm 0.07$) (Figure 1, Supplemental Figure S1A) and further, 1-5E exhibited higher cell detachment rate ($1.1 \times 10^8 \pm 1.0 \times 10^6$ CFU/mm^2/min, $p = 2.3 \times 10^{-32}$) (Figure 1, Supplemental Figure S1B) and lower EPS production rate (0.7-times, $p = 6.0 \times 10^{-8}$) (Figure 1, Supplemental Figure S2) compared to WT ($OD_{590} = 0.45 \pm 0.07$; cell detachment rate = $1.30 \times 10^8 \pm 1.0 \times 10^6$ CFU/mm^2/min). Other susceptible isolates 2-4G ($OD_{590} = 0.40 \pm 0.10$, $p = 0.24$) and 2-5C exhibited similar rates of biofilm biomass ($OD_{590} = 0.38 \pm 0.10$, $p = 0.10$) (Figure 1, Supplemental

Figure S1A) and cell detachment rates (2-4G: $1.2 \times 10^8 \pm 1.5 \times 10^{-7}$ CFU/mm^2/min, p = 0.05; 2-5C: $1.7 \times 10^8 \pm 5.3 \times 10^{-7}$ CFU/mm^2/min, p = 0.3) as that of WT (OD_{590} = 0.45 ± 0.07; cell detachment rate = $1.3 \times 10^8 \pm 1.0 \times 10^6$ CFU/mm^2/min) (Figure 1, Supplemental Figure S1B) in presence of H_2O_2. Mutant 2-4G exhibited significantly lower EPS production rate (0.37-times, p = 4×10^{-3}), whereas 2-5C (1.1-times, p = 0.24) displayed similar rates of EPS production as WT in the presence of H_2O_2 (Figure 1, Supplemental Figure S2).

SI-3.4: Oxidative response by biofilms of isolates with enhanced virulence traits (Class C)

Isolated with enhanced virulence traits reported considerably lower oxidative stress response in presence of H_2O_2. Isolates 1-10C (OD_{590} = 0.18 ± 0.12, p = 5.8×10^{-9}) and 1-11B (OD_{590} = 0.15 ± 0.05, p = 9.0×10^{-4}) retained 0.4-times and 0.3-times lower biofilm biomass (Figure 1, Supplemental Figure S1A), higher cell detachment rates (1-10C: $2.1 \times 10^8 \pm 1.4 \times 10^{-7}$, p = 1.3×10^{-17}; 1-11B: $2.0 \times 10^8 \pm 1.1 \times 10^{-7}$, p = 1.6×10^{-18}) (Figure 1, Supplemental Figure S1B), and 0.7-times lower EPS production rate ($p<0.05$) (Figure 1, Supplemental Figure S2) compared to WT upon induction of H_2O_2-mediated oxidative stress. Isolates 2-2B, 2-3G and 2-12C displayed similar rates of biofilm biomass (>0.8-times, $p>0.05$) (Figure 1, Supplemental Figure S1A) and cell detachment rates (2-2B: $1.1 \times 10^8 \pm 1.9 \times 10^{-7}$ CFU/mm^2/min p = 0.52; 2-3G: $1.5 \times 10^8 \pm 1.6 \times 10^{-7}$ CFU/mm^2/min, p = 0.68; 2-12C: $1.1 \times 10^8 \pm 2.5 \times 10^{-7}$ CFU/mm^2/min, p = 0.65) as that of WT (cell detachment rate = $1.3 \times 10^8 \pm 1.0 \times 10^6$ CFU/mm^2/min) (Figure 1, Supplemental Figure S1B). 2-3G and 2-12C generated 0.6-times (p = 2.33×10^{-3}) and 0.7-times lower (p = 8.02×10^{-3}) EPS than WT, while EPS production similar to WT was observed for 2-2B (p = 0.28) (Figure 1, Supplemental Figure S2).

SI-3.5: Oxidative response by biofilms of isolates with enhanced oxidative traits (Class D)

In the presence of H_2O_2, a significant increase in biofilm biomass retention coupled with lower cell detachment rates were observed for isolates 1-3B (OD_{590} = 0.70 ± 0.06, p = 1.80 x 10^{-8}); cell detachment rate = 8.20 x 10^7 ± 7.80 x 10^5 CFU/mm^2/min, p = 5.70 x 10^{-5} and 2-8D (OD_{590} = 0.95 ± 0.33, p = 0.013; cell detachment rate = 6.50 x 10^7 ± 1.20 x 10^7 CFU/mm^2/min, p = 4.70 x 10^{-4}) (Figure 1, Supplemental Figure S1A, S1B). In addition, 1.3-times higher EPS production was observed in the case of 1-3B (p = 1.3 x 10^{-7}) and 2-8D (p = 0.04) upon exposure to H_2O_2 (Figure 1, Supplemental Figure S2).

SI-3.6: Oxidative response by biofilms of isolates with enhanced motility and siderophore production (Class E)

2-7B retained 1.6-times more biofilm biomass (OD_{590} = 0.72 ± 0.13, p = 7.40 x 10^{-8}) (Figure 1, Supplemental Information-1: Figure S1A) with lower cell detachment rates (1.10 x 10^8 ± 1.20 x 10^7 CFU/mm^2/min, p = 0.02) (Figure 1, Supplemental Figure S1B) and similar EPS production rate (p = 0.34) (Figure 1, Supplemental Figure S2) relative to WT (OD_{590} = 0.45 ± 0.07; cell detachment rate = 1.30 x 10^8 ± 1.0 x 10^6 CFU/mm^2/min). On the other hand, H_2O_2-mediated oxidative stress did not induce any changes in the oxidative stress response of 1-7E (Biofilm Biomass: OD_{590} = 0.51 ± 0.09, p = 0.08; Cell detachment rate = 1.26 x 10^8 ± 1.33 x 10^7 CFU/mm^2/min, p = 0.70) (Figure 1, Supplemental Figure S1A, S2).

SI-3.7: Virulence factors exhibited by PI-7 WT and mutants

Virulence factors enable the bacterial cells to invade and colonize host cells. Colonizing host cells through biofilm formation by pathogens form the first step towards chronic pathogenic infections (O'Loughlin et al., 2013;Stępień-Pyśniak et al., 2019). Hence, PI-7 WT and mutants were tested for phenotypic differences in virulence factors related to biofilm formation (Supplemental Figure S3), siderophore production (Supplemental Figure S4), motility (Supplemental Figure S5), curli

production (Supplemental Figure S6) and internalization into mammalian cells (Supplemental Figure S7). The varying phenotypic traits displayed by PI-7 mutants relative to WT is shown in Figure 1.

SI-3.8: Virulence factors exhibited by resistant isolates (Class A)

Compared to WT, resistant isolates 1-2A and 2-7E exhibited higher biofilm production, motility, curli production and internalization into mammalian cells. 1-2A (OD_{590} = 1.13 ± 0.13, p = 5.29 x 10^{-12}) and 2-7E (OD_{590} = 1.06 ± 0.21, p = 3.56 x 10^{-8}) produced >2.0-times higher biofilm biomass than WT (OD_{590} = 0.50 ± 0.09) (Figure 1, Supplemental Figure S3). Siderophore production by 1-2A and 2-7E was found to be similar to WT in both M9 (1-2A: p = 0.46; 2-7E: p = 0.54) and M63 media (1-2A: p = 0.77; 2-7E: p = 0.63) (Figure 1, Supplemental Figure S4). 1-2A displayed 1.5-times (Swimming zone diameter = 14.50 ± 1.90 mm, p = 1.0 x 10^{-16}) and 1.6-times (Swarming zone diameter = 15.10 ± 2.0 mm, p = 3.50 x 10^{-7}) higher swimming and swarming motility relative to WT (Swimming zone diameter = 10.0 ± 0.30 mm; Swarming zone diameter = 9.60 ± 0.80 mm). 2-7E (Swimming zone diameter = 14.0 ± 0.50 mm; Swarming zone diameter = 14.30 ± 1.50 mm) also displayed significantly higher rates of swimming (1.4-times) and swarming (1.5-times) motility (Figure 1, Supplemental Figure S5). Significantly decreased amount of curli fibers were observed in the cell free supernatant for both 1-2A (p<0.05) and 2-7E (p<0.05) at different cell densities compared to WT, which implies to an increase in cell surface-localized curli production by both resistant isolates (Figure 1, Supplemental Figure S6). Similarly, 1-2A (A549 cells: 5.40 x 10^{-4} ± 1.10 x 10^{-4} CFU internalized/CFU inoculated/h, p = 0.01; HeLa cells: 8.40 x 10^{-3} ± 7.0 x 10^{-4} CFU internalized/CFU inoculated/h, p = 2.30 x 10^{-4}) and 2-7E (A549 cells: 5.30 x 10^{-4} ± 1. 40 x 10^{-4} CFU internalized/CFU inoculated/h, p = 0.04; HeLa cells: 5.50 x 10^{-3} ± 6.30 x 10^{-4} CFU internalized/CFU inoculated/h, p = 4.0 x 10^{-3}) also displayed significantly higher internalization

into mammalian cells compared to WT (A549 cells: $2.60 \times 10^{-4} \pm 2.60 \times 10^{-5}$ CFU internalized/CFU inoculated/h; HeLa cells: $2.50 \times 10^{-3} \pm 2.90 \times 10^{-4}$ CFU internalized/CFU inoculated/h) (Figure 1, Supplemental Figure S7).

SI-3.9: Virulence factors exhibited by susceptible isolates (Class B)

Considerable decrease in siderophore production and motility was observed for susceptible isolates 1-5E, 2-4G and 2-5C. Susceptible isolate 1-5E ($OD_{590} = 0.39 \pm 0.04$, $p = 2.40 \times 10^{-3}$) produced 0.8-times lower biofilm biomass, whereas 2-4G ($OD_{590} = 0.44 \pm 0.06$, $p = 0.50$) and 2-5C ($OD_{590} = 0.49 \pm 0.12$, $p = 0.94$) exhibited similar rates of biofilm formation as WT ($OD_{590} = 0.50 \pm 0.09$) (Figure 1, Supplemental Figure S3). Significantly lower rates of siderophore production was observed in the case of 1-5E (M9: 0.7-times, $p = 6.30 \times 10^{-12}$; M63: 0.4-times, $p = 9.90 \times 10^{-8}$), 2-4G (M9: 0.4-times, $p = 7.10 \times 10^{-9}$; M63: 0.4-times, $p = 3.40 \times 10^{-12}$), and 2-5C (M9: 0.6-times, $p = 1.70 \times 10^{-3}$; M63: 0.7-times, $p = 1.50 \times 10^{-3}$) (Figure 1, Supplemental Figure S4) relative to WT. Decreased rates of motility were observed for 1-5E (Swimming zone diameter = 7.70 ± 0.1, $p = 9.30 \times 10^{-16}$; Swarming zone diameter = 7.0 ± 0.20, $p = 2.10 \times 10^{-6}$), 2-4G (Swimming zone diameter = 8.30 ± 0.10, $p = 2.50 \times 10^{-9}$; Swarming zone diameter = 6.30 ± 0.50, $p = 2.0 \times 10^{-7}$), and 2-5C (Swimming zone diameter = 8.10 ± 0.50, $p = 5.40 \times 10^{-7}$; Swarming zone diameter = 6.60 ± 0.50, $p = 2.40 \times 10^{-10}$) compared to WT (Swimming zone diameter = 10.0 ± 0.30 mm; Swarming zone diameter = 9.60 ± 0.80 mm) (Figure 1, Supplemental Figure S5). Significantly decreased curli production was observed for 1-5E and 2-5C ($p<0.05$), however 2-4G ($p>0.05$) produced curli at similar rates to WT (Figure 1, Supplemental Figure S6). Diminished rates of mammalian cells-based adhesion were observed for 1-5E (A549 cells: $7.80 \times 10^{-5} \pm 7.40 \times 10^{-6}$ CFU internalized/CFU inoculated/h, $p = 1.90 \times 10^{-5}$; HeLa cells: $1.30 \times 10^{-3} \pm 3.70 \times 10^{-4}$ CFU internalized/CFU inoculated/h, $p = 0.03$) and 2-5C (A549 cells: $1.10 \times 10^{-4} \pm 3.50 \times 10^{-5}$ CFU

internalized/CFU inoculated/h, $p = 1.0 \times 10^{-3}$; HeLa cells: $1.50 \times 10^{-3} \pm 2.90 \times 10^{-4}$ CFU internalized/CFU inoculated/h, $p = 0.04$), while there was only a marginal decrease in mammalian cells-based adhesion for 2-4G (A549 cells: $1.70 \times 10^{-4} \pm 2.70 \times 10^{-5}$ CFU internalized/CFU inoculated/h, $p = 0.06$; HeLa cells: $2.10 \times 10^{-3} \pm 3.80 \times 10^{-4}$ CFU internalized/CFU inoculated/h, $p = 0.36$) (Figure 1, Supplemental Figure S7).

SI-3.10: Virulence factors exhibited by isolates with enhanced virulence traits (Class C)

Repression of oxidative stress response among the isolates 1-10C, 1-11B, 2-2B, 2-3G and 2-12C was complemented with a significant increase in biofilm formation, motility, siderophore and curli production as well as mammalian cells-based internalization rate. Significantly enhanced biofilm biomass was exhibited by isolates 1-10C ($OD_{590} = 0.83 \pm 0.17$, $p = 6.0 \times 10^{-6}$), 1-11B ($OD_{590} = 0.80 \pm 0.17$, $p = 3.60 \times 10^{-5}$), 2-2B ($OD_{590} = 0.71 \pm 0.09$, $p = 1.60 \times 10^{-5}$), 2-3G ($OD_{590} = 0.66 \pm 0.01$, $p = 5.10 \times 10^{-4}$) and 2-12C ($OD_{590} = 0.66 \pm 0.14$, $p = 4.30 \times 10^{-3}$) (Figure 1, Supplemental Figure S3). Similar trend was observed for siderophore production ($p<0.05$) in the case of isolates 1-10C, 1-11B, 2-2B, 2-3G and 2-12C (Figure 1, Supplemental Figure S4). Compared to WT (Swimming zone diameter = 10.0 ± 0.30 mm; Swarming zone diameter = 9.60 ± 0.80 mm), 1-10C (Swimming zone diameter = 14.40 ± 0.90 mm, $p = 2.80 \times 10^{-19}$; Swarming zone diameter = 13.70 ± 3.50 mm, $p = 6.0 \times 10^{-4}$), 1-11B (Swimming zone diameter = 14.40 ± 0.10 mm, $p = 1.20 \times 10^{-20}$; Swarming zone diameter = 15.0 ± 2.90 mm, $p = 3.60 \times 10^{-6}$), 2-2B (Swimming zone diameter = 15.60 ± 0.70 mm, $p = 2.40 \times 10^{-24}$; Swarming zone diameter = 15.40 ± 1.60 mm, $p = 6.10 \times 10^{-9}$), 2-3G (Swimming zone diameter = 13.70 ± 0.50 mm, $p = 7.0 \times 10^{-22}$; Swarming zone diameter = 15.20 ± 1.70 mm, $p = 5.60 \times 10^{-8}$), and 2-12C (Swimming zone diameter = 14.60 ± 0.50 mm, $p = 3. 70 \times 10^{-21}$; Swarming zone diameter = 13.60 ± 3.30 mm, $p = 4.0 \times 10^{-4}$) also displayed a considerable increase in swimming and swarming motilities (Figure 1, Supplemental Figure S5).

Curli production by 1-10C, 2-2B, 2-3G and 2-12C increased considerably compared to WT ($p<0.05$) (Figure 1, Supplemental Figure S6). There was also significant increase in internalization into mammalian cells by 1-10C (A549 cells: $4.70 \times 10^{-4} \pm 1.0 \times 10^{-4}$ CFU internalized/CFU inoculated/h, p = 0.04; HeLa cells: $5.40 \times 10^{-3} \pm 1.0 \times 10^{-4}$ CFU internalized/CFU inoculated/h, p = 0.04), 1-11B (A549 cells: $5.10 \times 10^{-4} \pm 4.60 \times 10^{-5}$ CFU internalized/CFU inoculated/h, p = 2.90×10^{-4}; HeLa cells: $5.90 \times 10^{-3} \pm 1.90 \times 10^{-4}$ CFU internalized/CFU inoculated/h, p = 0.04), 2-2B (A549 cells: $1.10 \times 10^{-3} \pm 1.50 \times 10^{-4}$ CFU internalized/CFU inoculated/h, p = 2.20×10^{-3}; HeLa cells: $8.30 \times 10^{-3} \pm 1.10 \times 10^{-3}$ CFU internalized/CFU inoculated/h, p = 0.04), 2-3G (A549 cells: $4.03 \times 10^{-4} \pm 3.93 \times 10^{-5}$ CFU internalized/CFU inoculated/h, p = 0.03; HeLa cells: $3.78 \times 10^{-3} \pm 9.27 \times 10^{-4}$ CFU internalized/CFU inoculated/h, p = 0.01) and 2-12C (A549 cells: $3.80 \times 10^{-4} \pm 3.20 \times 10^{-5}$ CFU internalized/CFU inoculated/h, p = 8.0×10^{-3}; HeLa cells: $3.80 \times 10^{-3} \pm 3.30 \times 10^{-4}$ CFU internalized/CFU inoculated/h, p = 0.02) compared to WT (A549 cells: $2.60 \times 10^{-4} \pm 2.60 \times 10^{-5}$ CFU internalized/CFU inoculated/h; HeLa cells: $2.50 \times 10^{-3} \pm 2.90 \times 10^{-4}$ CFU internalized/CFU inoculated/h) (Figure 1, Supplemental Figure S7).

SI-3.11: Virulence factors exhibited by isolates with enhanced oxidative traits (Class D)

Biofilm biomass displayed by 1-3B ($OD_{590} = 0.52 \pm 0.07$, p = 0.16) and 2-8D ($OD_{590} = 0.58 \pm 0.19$, p = 0.34) was comparable with WT ($OD_{590} = 0.50 \pm 0.09$) (Figure 1, Supplemental Figure S3). 1-3B produced 0.7-times (p = 1.92×10^{-5}) and 0.4-times (p = 6.21×10^{-9}) lower amount of siderophores, whereas 2-8D produced 1.7-times (p = 3.20×10^{-8}) and 2.0-times (p = 7.80×10^{-7}) higher amount of siderophores in in M63 and M9 media respectively (Figure 1, Supplemental Figure S4). 1-3B exhibited 0.8-times lower swimming (p = 2.52×10^{-4}) and swarming (p = 3.30×10^{-4}) motilities, whereas no change in motility was observed for 2-8D ($p>0.05$) (Figure 1, Supplemental Figure S5). Curli production by 1-3B and 2-8D were comparable with WT ($p>0.05$)

(Figure 1, Supplemental Figure S6). No significant change was observed for 1-3B with respect to internalization into mammalian cells (A549 cells internalization rate = 2.25 x 10^{-4} ± 2.75 x 10^{-5} CFU internalized/CFU inoculated/h, p = 0.08; HeLa cells internalization rate = 2.94 x 10^{-3} ± 2.56 x 10^{-4} CFU internalized/CFU inoculated/h) compared to WT (A549 cells internalization rate: 2.60 x 10^{-4} ± 2.60 x 10^{-5} CFU internalized/CFU inoculated/h; HeLa cells internalization rate: 2.50 x 10^{-3} ± 2.90 x 10^{-4} CFU internalized/CFU inoculated/h), while 2-8D showed a considerable decline in internalization into A549 (2-8D internalization rate = 1.20 x 10^{-4} ± 2.40 x 10^{-5} CFU internalized/CFU inoculated/h, p = 5.90 x 10^{-5}) and HeLa cells (2-8D internalization rate = 1.30 x 10^{-4} ± 1.80 x 10^{-5} CFU internalized/CFU inoculated/h, p = 0.01) (Figure 1, Supplemental Figure S7).

SI-3.12: Virulence factors exhibited by isolates with enhanced motility and siderophore production (Class E)

Class E isolates 1-7E and 2-7B displayed significant increase in siderophore production and motility traits with contrasting response to curli production. Increase in biofilm formation was observed for 1-7E (OD_{590} = 0.63 ± 0.19, p = 0.04) and 2-7B (OD_{590} = 0.70 ± 0.04, p = 2.02 x 10^{-6}) compared to WT (OD_{590} = 0.50 ± 0.09) (Figure 1, Supplemental Figure S3). Significant increase in siderophore production was observed for 1-7E (2.3-times in M9 ($p<0.05$) and 2.8-times in M63 media ($p<0.05$)) and 2-7B (2.10-times in M9 ($p<0.05$) and 2.7-times in M63 media ($p<0.05$)) (Figure 1, Supplemental Figure S4). Swimming and Swarming motility traits of 1-7E (Swimming zone diameter = 13.90 ± 1.0 mm, p = 3.20 x 10^{-23}; Swarming zone diameter = 13.80 ± 2.70 mm, p = 3.0 x 10^{-5}) and 2-7B (Swimming zone diameter = 14.70 ± 1.10 mm, p = 7.70 x 10^{-23}; Swarming zone diameter = 14.60 ± 1.40, p = 2.50 x 10^{-8}) were also found to be enhanced significantly (Figure 1, Supplemental Figure S5). 2-7B exhibited a significant increase in curli production ($p<0.05$)

(Figure 1, Supplemental Information-1: Figure S6). However, both 1-7E and 2-7B did not exhibit any change in internalization rate (p>0.05) onto A549 and HeLa cells (Figure 1, Supplemental Figure S7).

4. GENOMIC ANALYSIS OF ISOLATES

SI-4.1: Transposon insertions and deletions from *E. coli* PI-7 Random Transposon Insertion Site Library

A total of 105 distinct structural variant (SV) loci were discovered using PacBio long read resequencing of the 14 mutants, with an average of 74.6 present in each mutant. Specifically, 64 insertions, 14 deletions, 16 translocations, 7 duplications, 2 inversions, and 2 copy number variations were observed. 51 SVs are present at least heterozygotic in all 14 mutants (26 insertions, 8 deletions, 10 translocations, 4 duplications, 2 copy number variations, and 1 inversion). Three translocations are called homozygous in all mutants as well as one deletion, likely indicating mis-assembly of the reference. Out of 64 insertions, 28 insertion calls were shorter than 100 bp and consist of low complexity sequence, which are likely due to sequencing errors and the remaining 36 insertions were longer than 100 bp. Tn5 Transposon related insertion sequences detected using ISfinder (Siguier et al., 2006) ($e < 1e^{-10}$), which yielded yields 22 significant hits. The presence/absence of each insertion at the various loci is shown in Supplemental Figure S8A and B and detailed information for all 36 long insertions are provided in Supplemental Table S2 including the nearest gene up- and downstream from the insertion locus. Detailed information of gene overlapping with deletions are provided in Supplemental Table S3, while details of genes flanking the deletions are listed in Supplemental Table S4.

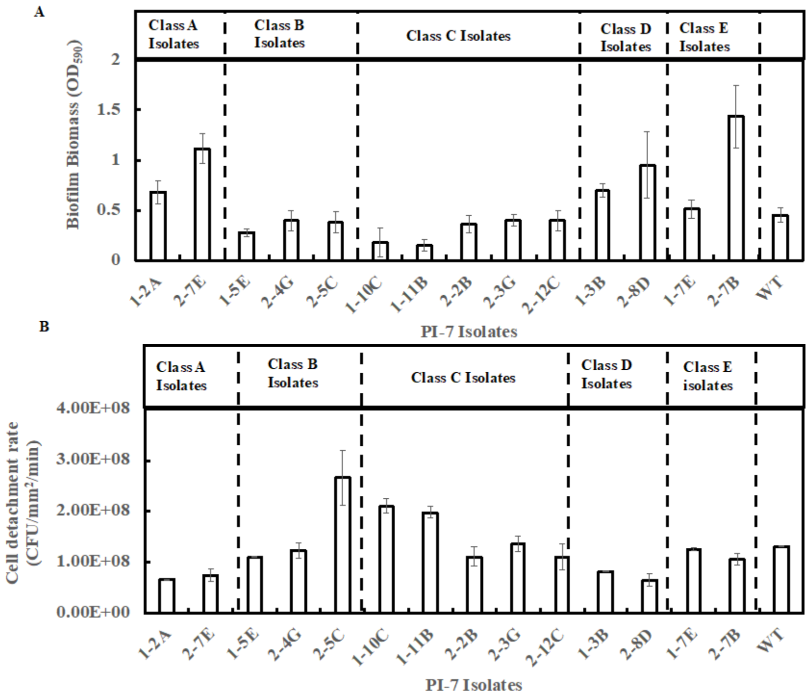

Figure S1: Response of PI-7 WT and mutant biofilms exposed to oxidative stress induced by exposure to H_2O_2 for 1h. (A) Effect of H_2O_2-induced oxidative stress on mature biofilms of PI-7 WT and mutants evaluated through biofilm biomass; (B) Effect of H_2O_2-induced oxidative stress on cell dispersal from biofilms of PI-7 WT and mutants evaluated through cell detachment rate. Results are presented as mean ± standard deviation (n=4)

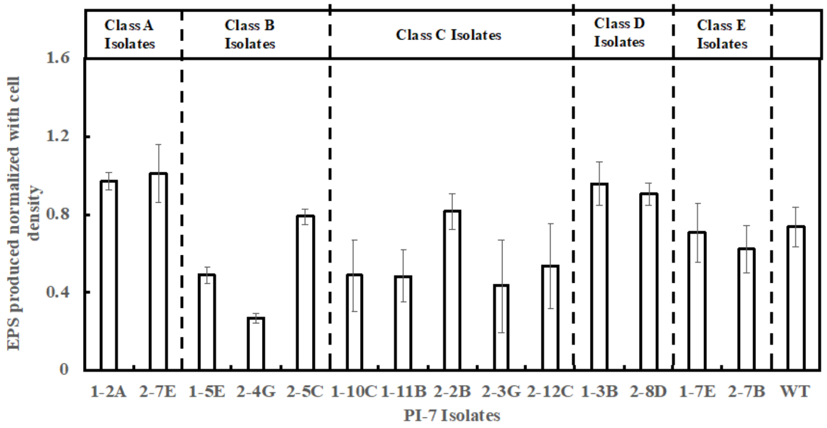

Figure S2: Effect of H_2O_2-induced oxidative stress on EPS production within biofilms of PI-7 WT and mutants. Results are presented as mean ± standard deviation (n=4).

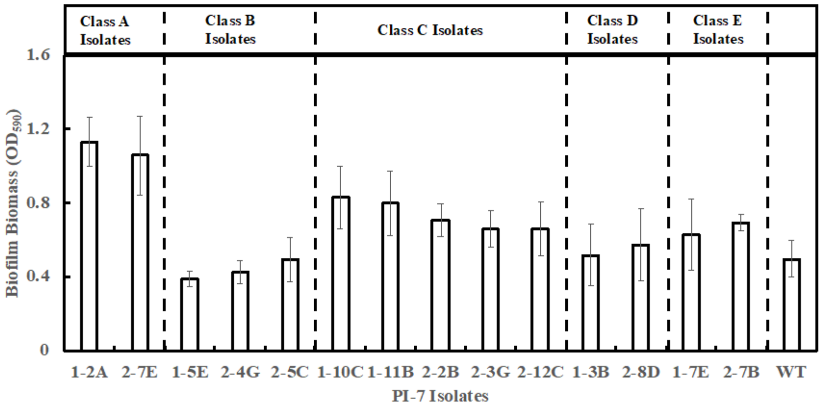

Figure S3: Biofilm formation exhibited by PI-7 WT and mutants. Results are presented as mean ± standard deviation (n=4).

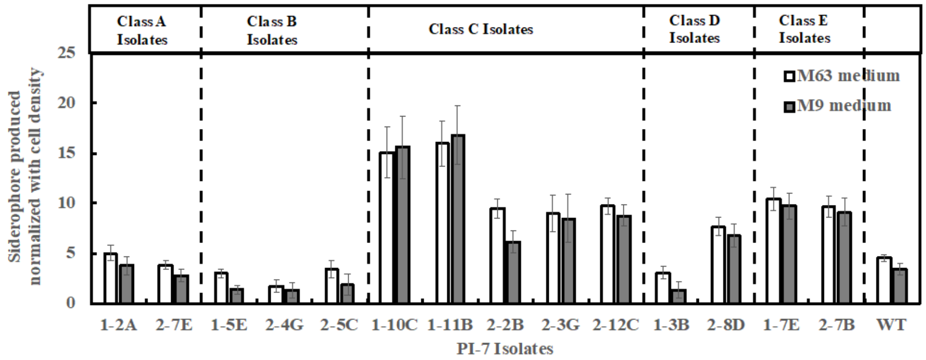

Figure S4: Siderophores produced by PI-7 WT and mutants. Siderophores produced by PI-7 isolates are normalized with cell density. Results are presented as mean ± standard deviation (n = 4)

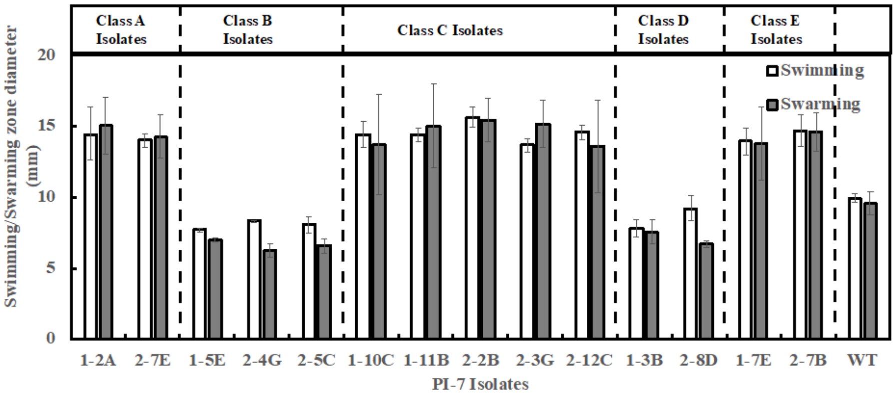

Figure S5: Swimming and swarming motility exhibited by PI-7 WT and mutants evaluated by the diameter of swimming and swarming zones. Results are presented as mean ± standard deviation (n = 12).

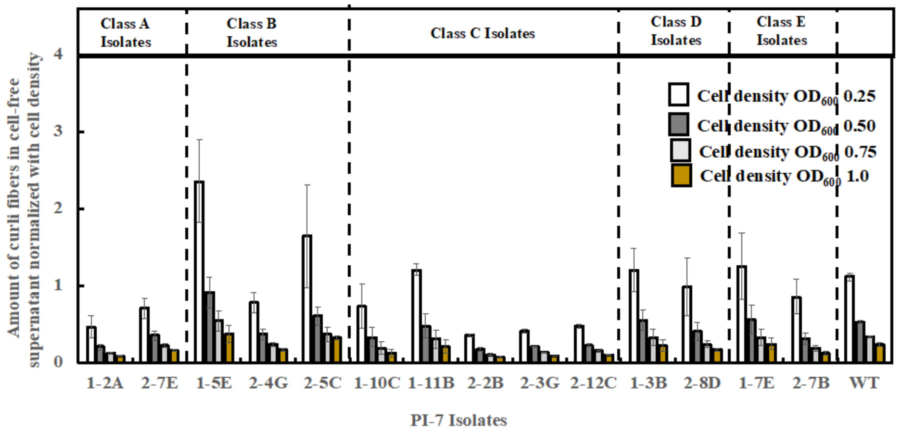

Figure S6: Relative amount of curli fibers released into cell exterior by PI-7 WT and mutants under varying cell densities. The amount of curli released is normalized with cell density. Decreased amount of curli in cell-free supernatant reflects enhanced degree of cell surface-associated curli production. Results are presented as mean ± standard deviation (n=12).

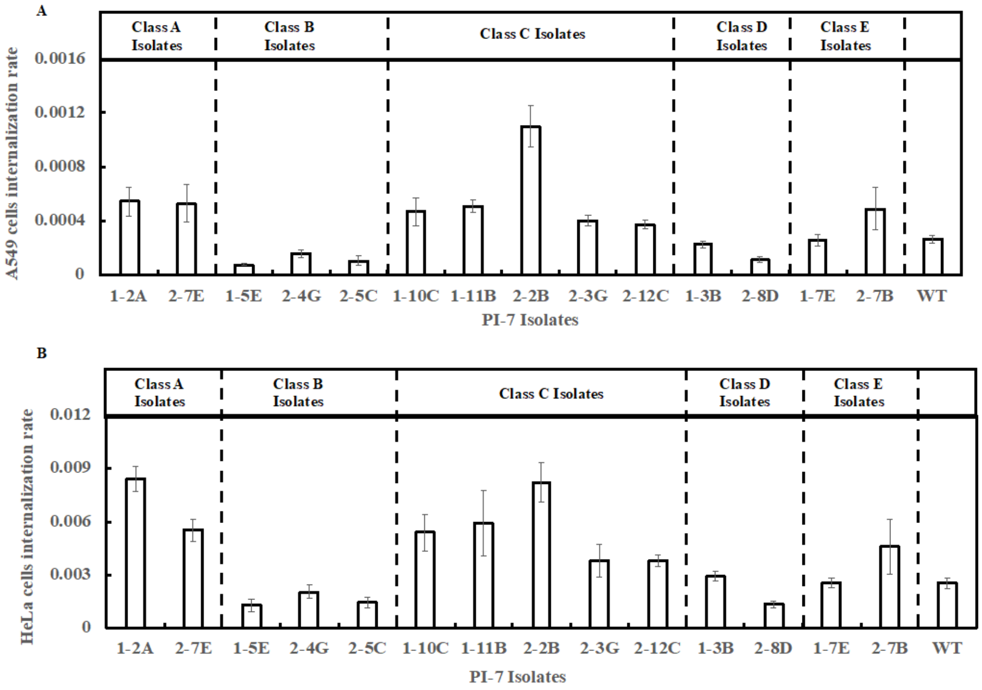

Figure S7: Internalization of PI-7 WT and mutants onto mammalian cells A-549 and HeLa cells. Extent of internalization is monitored in terms of cell internalization rate. (A). Internalization into A549 cells. (B) Internalization into HeLa cells. Results are presented as mean ± standard deviation (n=9).

A

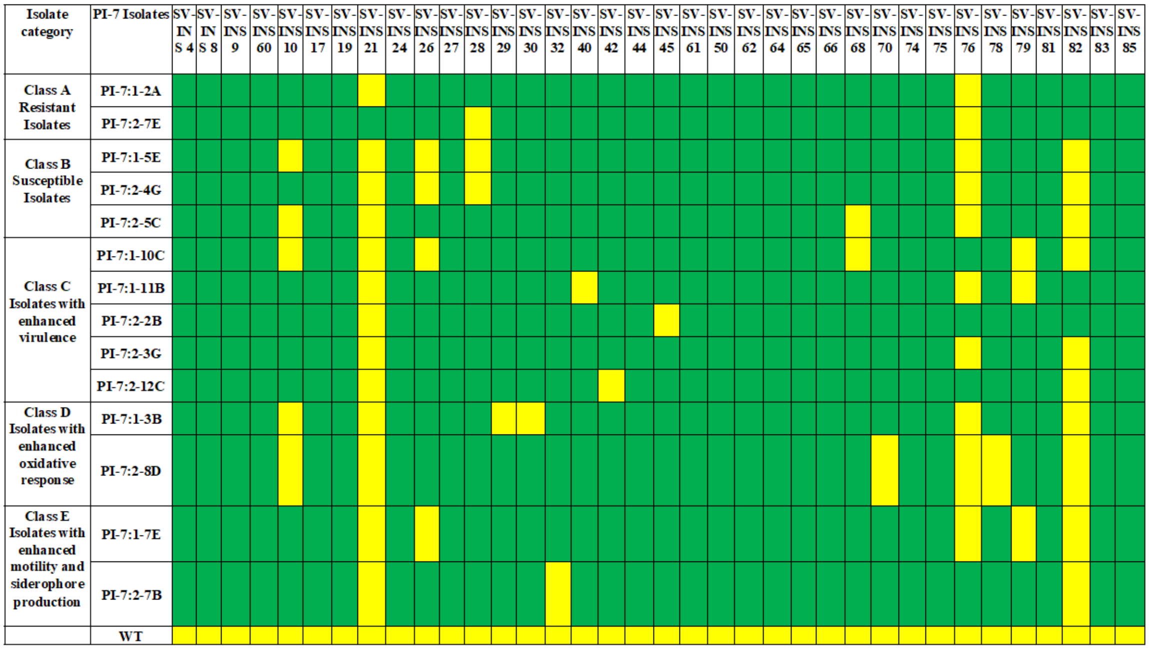

B

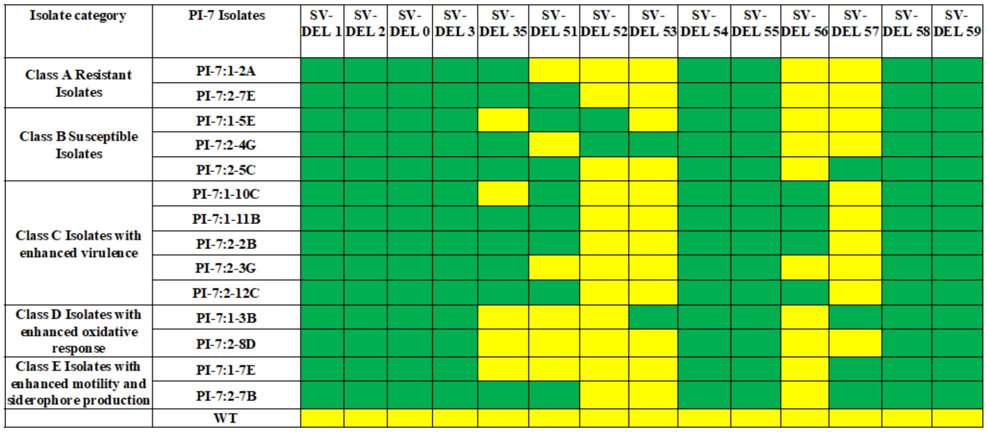

Figure S8: Genotype matrix representing transposon insertions and deletions in *E. coli* PI-7 random transposon mutants. Green shows the genetic alterations, while yellow indicates absence of any mutation (A) Details of genomic insertions in *E. coli* PI-7 random transposon mutants. SVINS is abbreviation for structural variation effected by genomic insertions and its details are listed in Supplemental Information-2: Supplemental Table S2 (B) Details of deletions in genome of *E. coli* PI-7 random transposon mutants. SVDEL is abbreviation for structural variation effected by genomic deletions and its details are listed in Supplemental Information-2: Supplemental Table S3 and Supplemental Table S4. Genomic alterations are shown in green, while yellow indicates the absence of respective insertion or deletion.

Table S1. Half-lives, lag-phase lengths and regression coefficients (R2) of disinfection-based inactivation kinetics of *E. coli* PI-7 WT and different categories of mutants upon exposure to chlorination and solar irradiation. P-value denotes comparison between that isolate's half-life value against that of control for that particular disinfection treatment.

**Chlorination**				
**Class of Isolates**	**Isolate**	**Half-life (min)**	**p-value**	**R^2**
Class A: Resistant Isolates	1-2A	10.9 ± 1.7	6.42×10^{-10}	0.99
	2-7E	9.5 ± 1.6	2.37×10^{-8}	0.98
Class B: Susceptible Isolates	1-5E	3.3 ± 0.1	1.18×10^{-11}	0.99
	2-4G	3.3 ± 0.05	1.32×10^{-13}	0.99
	2-5C	3.6 ± 0.06	1.09×10^{-11}	0.99
Class C: Virulent Isolates	1-10C	7.0 ± 0.08	1.08×10^{-11}	0.98
	1-11B	7.6 ± 0.3	1.06×10^{-9}	0.99
	2-2B	7.7 ± 0.2	1.10×10^{-10}	0.99
	2-3G	7.2 ± 0.2	1.09×10^{-9}	0.99
	2-12C	7.5 ± 0.2	1.72×10^{-11}	0.98
Class D: Isolates with enhanced oxidative stress response	1-3B	6.2 ± 0.14	1.35×10^{-6}	0.99
	2-8D	6.8 ± 0.6	2.34×10^{-5}	0.99
Class E: Isolates with enhanced motility and siderophore production	1-7E	5.3 ± 0.03	0.18	0.99
	2-7B	5.2 ± 0.05	0.80	0.99
**WT**		5.2 ± 0.2		0.99

**Solar Irradiation**						
**Class of Isolates**	**Isolate**	**Half-life (min)**	**p-value**	**Lag phase (h)**	**p-value**	**R^2**

Class A: Resistant Isolates	1-2A	7.8 ± 0.4	1.17×10^{-10}	7.0	5.54×10^{-7}	0.99
	2-7E	7.2 ± 2.0	1.79×10^{-5}	6.2 ± 0.1	0.04	0.98
Class B: Susceptible Isolates	1-5E	2.2 ± 0.03	2.57×10^{-11}	2.0	2.20×10^{-13}	0.95
	2-4G	3.6 ± 0.2	4.0×10^{-3}	3.3 ± 1.0	1.14×10^{-5}	0.92
	2-5C	4.2 ± 0.03	0.71	2.0	2.20×10^{-13}	0.96
Class C: Virulent Isolates	1-10C	7.9 ± 0.1	9.15×10^{-14}	3.7 ± 0.2	2.56×10^{-7}	0.96
	1-11B	7.2 ± 2.6	1.0×10^{-3}	5.3 ± 0.2	0.176	0.98
	2-2B	7.3 ± 1.7	5.3×10^{-6}	6.0	0.06	0.99
	2-3G	7.4 ± 1.8	8.51×10^{-5}	5.0	1.0×10^{-3}	0.90
	2-12C	7.4 ± 0.6	1.14×10^{-8}	3.7 ± 0.1	6.29×10^{-5}	0.90
Class D: Isolates with enhanced oxidative stress response	1-3B	3.7 ± 0.3	0.08	6.2 ± 0.7	0.10	0.94
	2-8D	4.2 ± 0.04	0.59	4.2 ± 0.2	3.51×10^{-5}	0.94
Class E: Isolates with enhanced motility and siderophore production	1-7E	12.03 ± 0.5	3.38×10^{-15}	5.1 ± 0.1	0.01	0.94
	2-7B	8.02 ± 1.6	2.03×10^{-7}	4.7 ± 0.2	0.17	0.99
WT		4.3 ± 0. 1		5.6 ± 0.3		0.86

Supplementary Table S2, S3 and S4 can be downloaded from this link:
https://drive.google.com/file/d/1IA5CfoH8SVbNV9nKC9AE62p2khZDcPyO/view?usp=sharing

REFERENCES

Al-Jassim, N., Mantilla-Calderon, D., Wang, T., and Hong, P.-Y. (2017). Inactivation and gene expression of a virulent wastewater Escherichia coli strain and the nonvirulent commensal Escherichia coli DSM1103 strain upon solar irradiation. *Environ Sci Technol* 51, 3649-3659.

Armbruster, C.E., Hodges, S.A., and Mobley, H.L. (2013). Initiation of swarming motility by Proteus mirabilis occurs in response to specific cues present in urine and requires excess L-glutamine. *J Bacteriol* 195, 1305-1319.

Carter, M.Q., Louie, J.W., Feng, D., Zhong, W., and Brandl, M.T. (2016). Curli fimbriae are conditionally required in Escherichia coli O157: H7 for initial attachment and biofilm formation. *Food Microbiol* 57, 81-89.

Chapman, M.R., Robinson, L.S., Pinkner, J.S., Roth, R., Heuser, J., Hammar, M., Normark, S., and Hultgren, S. (2002). Role of Escherichia coli curli operons in directing amyloid fiber formation. *Science J* 295, 851-855.

Chow, S., Gu, K., Jiang, L., and Nassour, A. (2011). Salicylic acid affects swimming, twitching and swarming motility in Pseudomonas aeruginosa, resulting in decreased biofilm formation. *J Microbiol Immunol Infect* 15, 22-29.

Gaddy, J.A., Tomaras, A.P., and Actis, L.A. (2009). The Acinetobacter baumannii 19606 OmpA protein plays a role in biofilm formation on abiotic surfaces and in the interaction of this pathogen with eukaryotic cells. *Infect Immun* 77, 3150-3160.

Gophna, U., Barlev, M., Seijffers, R., Oelschlager, T., Hacker, J., and Ron, E. (2001). Curli fibers mediate internalization of Escherichia coli by eukaryotic cells. *Infect Immun* 69, 2659-2665.

Haiko, J., and Westerlund-Wikström, B. (2013). The role of the bacterial flagellum in adhesion and virulence. *Biology J* 2, 1242-1267.

Josenhans, C., and Suerbaum, S. (2002). The role of motility as a virulence factor in bacteria. *Int J Med Microbiol* 291, 605-614.

Lamont, I.L., Beare, P.A., Ochsner, U., Vasil, A.I., and Vasil, M.L. (2002). Siderophore-mediated signaling regulates virulence factor production in Pseudomonas aeruginosa. *P Natl Acad Sci Usa* 99, 7072-7077.

Li, H (2018). Minimap2: pairwise alignment for nucleotide sequences. *J Bioinform* 34, 3094-3100.

Mantilla-Calderon, D., Jumat, M.R., Wang, T., Ganesan, P., Al-Jassim, N., and Hong, P.-Y. (2016). Isolation and characterization of NDM-positive Escherichia coli from municipal wastewater in Jeddah, Saudi Arabia. *Antimicrob Agents Ch* 60, 5223-5231.

Neilands, J., and Nakamura, K. (2017). "Detection, determination, isolation, characterization and regulation of microbial iron chelates," in *Handbook of Microbial Iron Chelates (1991).* CRC press), 1-14.

Neilands, J.B. (1981). Microbial iron compounds. *Annu Rev Biochem* 50, 715-731.

O'loughlin, C.T., Miller, L.C., Siryaporn, A., Drescher, K., Semmelhack, M.F., and Bassler, B.L. (2013). A quorum-sensing inhibitor blocks Pseudomonas aeruginosa virulence and biofilm formation. *P Natl Acad Sci Usa* 110, 17981-17986.

Ramanan, N., and Wang, Y. (2000). A high-affinity iron permease essential for Candida albicans virulence. *Science J* 288, 1062-1064.

Rubens, C., Smith, S., Hulse, M., Chi, E., and Van Belle, G. (1992). Respiratory epithelial cell invasion by group B streptococci. *Infect Immun* 60, 5157-5163.
Saldaña, Z., Xicohtencatl-Cortes, J., Avelino, F., Phillips, A.D., Kaper, J.B., Puente, J.L., and Girón, J.A. (2009). Synergistic role of curli and cellulose in cell adherence and biofilm formation of attaching and effacing Escherichia coli and identification of Fis as a negative regulator of curli. *Environ Microbiol* 11, 992-1006.
Siguier, P., Pérochon, J., Lestrade, L., Mahillon, J., and Chandler, M. (2006). ISfinder: the reference centre for bacterial insertion sequences. *Nucleic Acids Res* 34, D32-D36.
Sivakumar, K., Mukherjee, M., Cheng, H.I., Zhang, Y., Ji, L., and Cao, B. (2015). Surface display of roGFP for monitoring redox status of extracellular microenvironments in Shewanella oneidensis biofilms. *Biotechnol Bioeng* 112, 512-520.
Sivakumar, K., Scarascia, G., Zaouri, N., Wang, T., Kaksonen, A.H., and Hong, P.-Y. (2019). Salinity-Mediated Increment in Sulfate Reduction, Biofilm Formation, and Quorum Sensing: A Potential Connection Between Quorum Sensing and Sulfate Reduction? *Front Microbiol* 10.
Stępień-Pyśniak, D., Hauschild, T., Kosikowska, U., Dec, M., and Urban-Chmiel, R. (2019). Biofilm formation capacity and presence of virulence factors among commensal Enterococcus spp. from wild birds. *Sci Rep* 9, 11204.
Taylor, K.A. (1995). A modification of the phenol/sulfuric acid assay for total carbohydrates giving more comparable absorbances. *Appl Biochem Biotechnol* 53, 207-214.
